# Accelerated Optimization on Riemannian Manifolds via Discrete Constrained Variational Integrators

**DOI:** 10.1007/s00332-022-09795-9

**Published:** 2022-04-28

**Authors:** Valentin Duruisseaux, Melvin Leok

**Affiliations:** Department of Mathematics, University of California, San Diego, La Jolla, CA 92093-0112 USA

**Keywords:** Riemannian optimization, Accelerated optimization, Symplectic optimization, Constrained variational integrators, 34A26, 37M15, 37N40, 65K10, 65P10, 70H45

## Abstract

A variational formulation for accelerated optimization on normed vector spaces was recently introduced in Wibisono et al. (PNAS 113:E7351–E7358, 2016), and later generalized to the Riemannian manifold setting in Duruisseaux and Leok (SJMDS, 2022a). This variational framework was exploited on normed vector spaces in Duruisseaux et al. (SJSC 43:A2949–A2980, 2021) using time-adaptive geometric integrators to design efficient explicit algorithms for symplectic accelerated optimization, and it was observed that geometric discretizations which respect the time-rescaling invariance and symplecticity of the Lagrangian and Hamiltonian flows were substantially less prone to stability issues, and were therefore more robust, reliable, and computationally efficient. As such, it is natural to develop time-adaptive Hamiltonian variational integrators for accelerated optimization on Riemannian manifolds. In this paper, we consider the case of Riemannian manifolds embedded in a Euclidean space that can be characterized as the level set of a submersion. We will explore how holonomic constraints can be incorporated in discrete variational integrators to constrain the numerical discretization of the Riemannian Hamiltonian system to the Riemannian manifold, and we will test the performance of the resulting algorithms by solving eigenvalue and Procrustes problems formulated as optimization problems on the unit sphere and Stiefel manifold.

## Introduction

Many data analysis algorithms are designed around the minimization of a loss function or the maximization of a likelihood function. Due to the ever-growing scale of the data sets and size of the problems, there has been a lot of focus on first-order optimization algorithms because of their low cost per iteration. In 1983, Nesterov’s accelerated gradient method (Nesterov 1983) was shown to converge in $\mathcal {O}(1/k^2)$ to the minimum of the convex objective function *f*, improving on the $\mathcal {O}(1/k)$ convergence rate exhibited by standard gradient descent methods. This $\mathcal {O}(1/k^2)$ convergence rate was shown in Nesterov (2004) to be optimal among first-order methods using only information about $\nabla f$ at consecutive iterates. This phenomenon in which an algorithm displays this improved rate of convergence is referred to as acceleration, and other accelerated algorithms have been derived since Nesterov’s algorithm, which was shown in Su et al. (2016) to limit to a second-order ordinary differential equation (ODE), as the timestep goes to 0, and that *f*(*x*(*t*)) converges to its optimal value at a rate of $\mathcal {O}(1/t^2)$ along any trajectory *x*(*t*) of this ODE. It was then shown in Wibisono et al. (2016) that in continuous time, an arbitrary convergence rate $\mathcal {O}(1/t^p)$ can be achieved in normed spaces, by considering flow maps generated by a family of time-dependent Bregman Lagrangian and Hamiltonian systems which is closed under time-rescaling. This variational framework and the time-rescaling property of this family were then exploited in Duruisseaux et al. (2021) by using time-adaptive geometric integrators to design efficient explicit algorithms for symplectic accelerated optimization. It was observed that a careful use of adaptivity and symplecticity could result in a significant gain in computational efficiency. More generally, when applied to Hamiltonian systems, symplectic integrators yield discrete approximations of the flow that preserve the symplectic 2-form (Hairer et al. 2006). The preservation of symplecticity results in the preservation of many qualitative aspects of the underlying dynamical system. In particular, when applied to conservative Hamiltonian systems, symplectic integrators exhibit excellent long-time near-energy preservation (Benettin and Giorgilli 1994; Reich 1999). Variational integrators provide a systematic method for constructing symplectic integrators of arbitrarily high-order based on the numerical discretization of Hamilton’s principle (Marsden and West 2001; Hall and Leok 2015), or equivalently, by the approximation of Jacobi’s solution of the Hamilton–Jacobi equation, which is a generating function for the exact symplectic flow map.

In the past few years, there has been some effort to derive accelerated optimization algorithms in the Riemannian manifold setting (Duruisseaux and Leok 2022a; Alimisis et al. 2020a, b, 2021; Zhang and Sra 2016, 2018; Ahn and Sra 2020; Liu et al. 2017). In Duruisseaux and Leok (2022a), it was shown that in continuous time, the convergence rate of *f*(*x*(*t*)) to its optimal value can be accelerated to an arbitrary convergence rate $\mathcal {O}(1/t^p)$ on Riemannian manifolds. This was achieved by considering a family of time-dependent Bregman Lagrangian and Hamiltonian systems on Riemannian manifolds which is closed under time-rescaling, thereby generalizing the variational framework for accelerated optimization of Wibisono et al. (2016) to Riemannian manifolds. The time-adaptivity based approach relying on a Poincaré transformation from Duruisseaux et al. (2021) was also extended to the Riemannian manifold setting in Duruisseaux and Leok (2022a). Now, the Whitney embedding theorems (Whitney 1944a, b) state that any smooth manifold of dimension $n \ge 2$ can be embedded in $\mathbb {R}^{2n}$ and immersed in $\mathbb {R}^{2n-1}$, and is thus diffeomorphic to a submanifold of $\mathbb {R}^{2n}$. Furthermore, the Nash embedding theorems (Nash 1956) state that any Riemannian manifold can be globally isometrically embedded into some Euclidean space. As a consequence of these embedding theorems, the study of Riemannian manifolds can in principle be reduced to the study of submanifolds of Euclidean spaces. Altogether, this motivates the introduction of time-adaptive variational integrators on Riemannian manifolds which exploit the structure of the embedding Euclidean space, and in this paper, we will study how holonomic constraints can be incorporated into different types of variational integrators to constrain the numerical solutions of the Riemannian dynamical system to the Riemannian manifold. Incorporating holonomic constraints in geometric integrators has been studied extensively in the past (see Marsden and West (2001); Hairer et al. (2006); Marsden and Ratiu (1999); Holm et al. (2009) for instance), and some work has been done from the variational perspective for the Type I Lagrangian formulation in Marsden and West (2001) via augmented Lagrangians.

### Outline of the Paper

Section 2 shows the equivalence between constrained variational principles and constrained Euler–Lagrange equations, both in continuous and discrete time, before deriving analogous results for both the Type II and Type III Hamiltonian formulations of mechanics in Sect. 3. In Sect. 4, we will exploit error analysis theorems for unconstrained mechanics from Marsden and West (2001); Schmitt and Leok (2017) to obtain variational error analysis results for the maps defined implicitly by the discrete constrained Euler–Lagrange and Hamilton’s equations. Finally, in Sect. 5, we will exploit these constrained variational integrators and the variational formulation of accelerated optimization on Riemannian manifolds from Duruisseaux and Leok (2022a) to solve numerically generalized eigenvalue problems and Procrustes problems on the unit sphere and Stiefel manifold.

## Constrained Variational Lagrangian Mechanics

Traditionally, variational integrators have been designed based on the Type I generating function known as the **discrete Lagrangian**, $L_d:\mathcal {Q} \times \mathcal {Q} \rightarrow \mathbb {R}$. The exact discrete Lagrangian is the exact generating function for the time-*h* flow map of Hamilton’s equations, and it can be represented in boundary value form by

2.1 $$\begin{aligned} L_d^E(q_0,q_h)=\int _0^h L(q(t),\dot{q}(t)) dt , \end{aligned}$$

where $q(0)=q_0,$ $q(h)=q_h,$ and *q* satisfies the Euler–Lagrange equations over the time interval [0, *h*]. This is closely related to Jacobi’s solution of the Hamilton–Jacobi equation. A variational integrator is defined by constructing an approximation $L_d:\mathcal {Q} \times \mathcal {Q} \rightarrow \mathbb {R}$ to the exact discrete Lagrangian $L_d^E$, and then applying the **implicit discrete Euler–Lagrange equations**,

2.2 $$\begin{aligned} p_0=-D_1 L_d(q_0, q_1),\qquad p_1=D_2 L_d(q_0, q_1), \end{aligned}$$

which implicitly define a numerical integrator, referred to as the **discrete Hamiltonian map** $\tilde{F}_{L_d}:(q_0,p_0)\mapsto (q_1,p_1)$, where $D_i$ denotes a partial derivative with respect to the *i*-th argument. These equations define the **discrete Legendre transforms**, $\mathbb {F}^{\pm }L_{d}: \mathcal {Q}\times \mathcal {Q} \rightarrow T^{*}\mathcal {Q}$:

2.3 $$\begin{aligned} \mathbb {F}^{+}L_{d}&:(q_{0},q_{1}) \mapsto (q_{1},p_{1}) = (q_{1},D_{2}L_{d}(q_{0},q_{1})), \end{aligned}$$

2.4 $$\begin{aligned} \mathbb {F}^{-}L_{d}&:(q_{0},q_{1}) \mapsto (q_{0},p_{0}) = (q_{0},-D_{1}L_{d}(q_{0},q_{1})), \end{aligned}$$

and the discrete Hamiltonian map can be expressed as $\tilde{F}_{L_d}\equiv (\mathbb {F}^{+}L_{d})\circ (\mathbb {F}^{-}L_{d})^{-1}$. Such numerical methods are called variational integrators as they can be derived from a **discrete Hamilton’s principle**, which involves extremizing a discrete action sum $S_d\left( \{q_k\}_{k=0}^N \right) \equiv \sum _{k=0}^{N-1} L_d(q_k,q_{k+1})$, subject to fixed boundary conditions on $q_0$, $q_N$.

Now, suppose we are given a configuration manifold $\mathcal {M}$, and a holonomic constraint function $\mathcal {C} : \mathcal {M} \rightarrow \mathbb {R}^d$. Assuming that $0\in \mathbb {R}^d$ is a regular point of $\mathcal {C}$, we can constrain the dynamics to the constraint submanifold $\mathcal {Q} = \mathcal {C}^{-1}(0),$ which is truly a submanifold of $\mathcal {M}$ (see Marsden and West (2001); Abraham et al. (1988)). We will now consider variational Lagrangian mechanics with holonomic constraints $\mathcal {C}(q)$ using Lagrange multipliers $\lambda : [0,T] \rightarrow \Lambda$.

### Continuous Constrained Variational Lagrangian Mechanics

We begin by presenting an equivalence between the continuous constrained variational principle and the continuous constrained Euler–Lagrange equations:

#### Theorem 2.1

Consider the **constrained action functional** $\mathfrak {S} : C^2([0,T], \mathcal {Q} \times \Lambda ) \rightarrow \mathbb {R}$ given by

2.5 $$\begin{aligned} \mathfrak {S} (q(\cdot ), \lambda (\cdot )) = \int _{0}^{T}{ \left[ L(q(t),\dot{q}(t) ) - \langle \lambda (t) , \mathcal {C}(q(t)) \rangle \right] dt}. \end{aligned}$$

The condition that $\mathfrak {S} (q(\cdot ), \lambda (\cdot ))$ is stationary with respect to the boundary conditions $\delta q(0) = 0$ and $\delta q(T) =0$ is equivalent to $\left( q(\cdot ),\lambda (\cdot ) \right)$ satisfying the **constrained Euler–Lagrange equations**

2.6 $$\begin{aligned} \frac{\partial L}{\partial q} - \frac{d}{dt} \frac{\partial L}{\partial \dot{q}} = \langle \lambda , \nabla \mathcal {C} (q) \rangle , \qquad \mathcal {C}(q) =0 . \end{aligned}$$

#### Proof

See Appendix A.1. $$\square $$

#### Remark 2.1

These constrained Euler–Lagrange equations can be thought of as the Euler–Lagrange equations coming from the augmented Lagrangian $\bar{L} \left( q,\lambda , \dot{q}, \dot{\lambda } \right) = L(q,\dot{q} )- \langle \lambda , \mathcal {C} (q) \rangle$.

Consider the function $\mathcal {S}(q_0,q_T)$ given by the extremal value of the constrained action functional $\mathfrak {S}$ over the family of curves $(q(\cdot ) , \lambda (\cdot ))$ satisfying the boundary conditions $q(0)=q_0$ and $q(T)=q_T$:

2.7 $$\begin{aligned} \mathcal {S}(q_0,q_T) = \mathop {\mathrm {ext}}\limits _{ \begin{array}{c} (q,\lambda )\in C^2([0,T],\mathcal {Q} \times \Lambda ) \\ q(0)=q_0, \quad q(T)=q_T \end{array}}{\mathfrak {S} (q(\cdot ) , \lambda (\cdot )) }. \end{aligned}$$

The following theorem shows that $\mathcal {S}(q_0,q_T)$ is a generating function for the flow of the continuous constrained Euler–Lagrange equations:

#### Theorem 2.2

The exact time-*T* flow map of Hamilton’s equations $(q_0,p_0) \mapsto (q_T,p_T)$ is implicitly given by the following relations:

2.8 $$\begin{aligned} D_1 \mathcal {S} (q_0,q_T) = - \frac{\partial L}{ \partial \dot{q}}(q_0,\dot{q}(0)), \qquad D_2 \mathcal {S} (q_0,q_T) = \frac{\partial L}{ \partial \dot{q}}(q_T,\dot{q}(T)) . \end{aligned}$$

In particular, $\mathcal {S} (q_0,q_T)$ is a Type I generating function that generates the exact flow of the constrained Euler–Lagrange equations (2.6).

#### Proof

See Appendix A.4.$$\square $$

### Discrete Constrained Variational Lagrangian Mechanics

We now introduce a discrete variational formulation of Lagrangian mechanics which includes holonomic constraints. Suppose we are given a partition $0 = t_0< t_1< \ldots < t_N = T$ of the interval [0, *T*], and a discrete curve in $\mathcal {Q} \times \Lambda$ denoted by $\{ (q_k, \lambda _k) \}_{k=0}^{N}$ such that $q_k \approx q(t_k)$ and $\lambda _k \approx \lambda (t_k)$. We will formulate discrete constrained variational Lagrangian mechanics in terms of the following discrete analogues of the constrained action functional $\mathfrak {S}$ given by Eq. (2.5):

2.9 $$\begin{aligned} \mathfrak {S}_d^+ \left( \{ (q_k, \lambda _k) \}_{k=0}^{N} \right)&= \sum _{k=0}^{N-1}{\left[ L_d(q_k,q_{k+1} )- \langle \lambda _{k+1} , \mathcal {C} (q_{k+1}) \rangle \right] }, \end{aligned}$$

2.10 $$\begin{aligned} \mathfrak {S}_d^- \left( \{ (q_k, \lambda _k) \}_{k=0}^{N} \right)&= \sum _{k=0}^{N-1}{\left[ L_d(q_k,q_{k+1} )- \langle \lambda _k , \mathcal {C} (q_k) \rangle \right] }, \end{aligned}$$

where

2.11 $$\begin{aligned} L_d(q_k,q_{k+1} )&\approx \mathop {\mathrm {ext}}\limits _{ \begin{array}{c} (q,\lambda )\in C^2([t_k,t_{k+1}],\mathcal {Q} \times \Lambda ) \\ q(t_{k})=q_{k}, \quad q(t_{k+1})=q_{k+1} \end{array}} \text { } \int _{t_k}^{t_{k+1}}{L(q(t),\dot{q}(t)) dt}. \end{aligned}$$

We can now derive discrete analogues to Theorem 2.1 relating discrete Type I variational principles to discrete Euler–Lagrange equations:

#### Theorem 2.3

The Type I discrete Hamilton’s variational principles

2.12 $$\begin{aligned} \delta \mathfrak {S}_d^{\pm } \left( \{ (q_k, \lambda _k) \}_{k=0}^{N} \right) = 0 \end{aligned}$$

are equivalent to the **discrete constrained Euler–Lagrange equations**

2.13 $$\begin{aligned} D_1 L_d(q_k,q_{k+1}) + D_2 L_d(q_{k-1},q_{k}) = \langle \lambda _k ,\nabla \mathcal {C}(q_k) \rangle , \quad \mathcal {C}(q_{k}) =0, \end{aligned}$$

where $$L_d(q_{k},q_{k+1}) $$ is defined via Eq. (2.11).

#### Proof

See Appendix A.7. $$\square $$

#### Remark 2.2

These discrete constrained Euler–Lagrange equations can be thought of as the discrete Euler–Lagrange equations coming from the augmented discrete Lagrangians

2.14 $$\begin{aligned} \bar{L}_d^+ \left( q_k,\lambda _k, q_{k+1}, \lambda _{k+1}\right)&= L_d(q_k,q_{k+1} )- \langle \lambda _{k+1} , \mathcal {C} (q_{k+1}) \rangle , \end{aligned}$$

2.15 $$\begin{aligned} \bar{L}_d^- \left( q_k,\lambda _k, q_{k+1}, \lambda _{k+1}\right)&= L_d(q_k,q_{k+1} )- \langle \lambda _k , \mathcal {C} (q_k) \rangle . \end{aligned}$$

## Constrained Variational Hamiltonian Mechanics

The boundary value formulation of the exact Type II generating function of the time-*h* flow of Hamilton’s equations is given by the exact discrete right Hamiltonian,

3.1 $$\begin{aligned} H_d^{+,E}(q_0,p_h) = p_h q_h - \int _0^h \left[ p(t) \dot{q}(t)-H(q(t), p(t)) \right] dt, \end{aligned}$$

where (*q*, *p*) satisfies Hamilton’s equations with boundary conditions $q(0)=q_0$ and $p(h)=p_h$. A Type II Hamiltonian variational integrator is constructed by using an approximate discrete right Hamiltonian $H_d^+$, and applying the **discrete right Hamilton’s equations**,

3.2 $$\begin{aligned} p_0=D_1H_d^+(q_0,p_1), \qquad q_1=D_2H_d^+(q_0,p_1), \end{aligned}$$

which implicitly define the integrator, $\tilde{F}_{H_d^+}:(q_0,p_0) \mapsto (q_1,p_1)$.

Similarly, the boundary value formulation of the exact Type III generating function of the time-*h* flow of Hamilton’s equations is given by the exact discrete left Hamiltonian,

3.3 $$\begin{aligned} H_d^{-,E}(q_h,p_0) = - p_0 q_0 - \int _0^h \left[ p(t) \dot{q}(t)-H(q(t), p(t)) \right] dt , \end{aligned}$$

where (*q*, *p*) satisfies Hamilton’s equations with boundary conditions $q(h)=q_h$ and $p(0)=p_0$. A Type III Hamiltonian variational integrator is constructed by using an approximate discrete left Hamiltonian $H_d^-$, and applying the **discrete left Hamilton’s equations**,

3.4 $$\begin{aligned} p_1= - D_1H_d^-(q_1,p_0), \qquad q_0 = - D_2H_d^-(q_1,p_0), \end{aligned}$$

which implicitly define the integrator, $\tilde{F}_{H_d^-}:(q_0,p_0) \mapsto (q_1,p_1)$.

We now derive analogous results to those of Sect. 2 from the Hamiltonian perspective. As in the Lagrangian case, we will assume we have a configuration manifold $\mathcal {M}$, a holonomic constraint function $\mathcal {C} : \mathcal {M} \rightarrow \mathbb {R}^d$, and that the dynamics are constrained to the submanifold $\mathcal {Q} = \mathcal {C}^{-1}(0)$.

### Continuous Constrained Variational Hamiltonian Mechanics

The following theorem presents the equivalence between a continuous constrained variational principle and continuous constrained Hamilton’s equations in the Type II case, generalizing Lemma 2.1 from Leok and Zhang (2011) to include holonomic constraints:

#### Theorem 3.1

Consider the Type II **constrained action functional** $\mathfrak {S} : C^2([0,T],T^*\mathcal {Q} \times \Lambda ) \rightarrow \mathbb {R}$

3.5 $$\begin{aligned} \mathfrak {S} (q(\cdot ),p(\cdot ), \lambda (\cdot ))= & {} p(T)q(T) - \int _{0}^{T}\left[ p(t) \dot{q}(t) - H(q(t),p(t))\right. \nonumber \\&\left. - \langle \lambda (t) , \mathcal {C}(q(t)) \rangle \right] dt. \end{aligned}$$

The condition that $\mathfrak {S} (q(\cdot ),p(\cdot ), \lambda (\cdot ))$ is stationary with respect to the boundary conditions $\delta q(0) = 0$ and $\delta p(T) =0$ is equivalent to $(q(\cdot ),p(\cdot ),\lambda (\cdot ))$ satisfying **Hamilton’s constrained equations**

3.6 $$\begin{aligned} \dot{q}= & {} \frac{\partial H}{\partial p} (q,p) , \qquad \dot{p} = -\frac{\partial H}{\partial q} (q,p) - \langle \lambda , \nabla \mathcal {C} (q) \rangle , \qquad \mathcal {C}(q) = 0 . \end{aligned}$$

#### Proof

See Appendix A.2. $$\square $$

As in the Type II case, we can derive a theorem relating a continuous constrained variational principle and continuous constrained Hamilton’s equations in the Type III case:

#### Theorem 3.2

Consider the Type III **constrained action functional** $\mathfrak {S} : C^2([0,T],T^*\mathcal {Q} \times \Lambda ) \rightarrow \mathbb {R}$

3.7 $$\begin{aligned}&\mathfrak {S} (q(\cdot ),p(\cdot ), \lambda (\cdot )) = - p(0)q(0) - \int _{0}^{T} \left[ p(t) \dot{q}(t) - H(q(t),p(t))\right. \nonumber \\&\left. - \langle \lambda (t) , \mathcal {C}(q(t)) \rangle \right] dt. \end{aligned}$$

The condition that $\mathfrak {S} (q(\cdot ),p(\cdot ), \lambda (\cdot ))$ is stationary with respect to the boundary conditions $\delta q(T) = 0$ and $\delta p(0) =0$ is equivalent to $(q(\cdot ),p(\cdot ),\lambda (\cdot ))$ satisfying **Hamilton’s constrained equations**

3.8 $$\begin{aligned} \dot{q} = \frac{\partial H}{\partial p} (q,p) , \qquad \dot{p} = -\frac{\partial H}{\partial q} (q,p) - \langle \lambda , \nabla \mathcal {C} (q) \rangle , \qquad \mathcal {C}(q) = 0 . \end{aligned}$$

#### Proof

See Appendix A.3. $$\square $$

#### Remark 3.1

Hamilton’s constrained equations are the same in the Type II and Type III formulations of Hamiltonian mechanics, and they can be thought of as the Hamilton’s equations generated by the augmented Hamiltonian3.9where  is the conjugate momentum for the variable $\lambda$. Furthermore, they are equivalent to the constrained Euler–Lagrange Eq. (2.6), provided that the Lagrangian *L* is hyperregular.

#### Remark 3.2

It is sometimes beneficial to augment the continuous equations with the equation $\langle \frac{\partial H}{\partial p} (q,p) , \nabla \mathcal {C}(q) \rangle =0$ (and analogously for the discrete case) to ensure that the momentum *p* lies in the cotangent space to the manifold, as explained and illustrated in (Hairer et al. 2006, Chapter VII).

We will now generalize Theorem 2.2 and its Type III analogue from Leok and Zhang (2011) to include holonomic constraints $\mathcal {C}(q)$ using Lagrange multipliers $\lambda : [0,T] \rightarrow \Lambda$.

In the Type II case, consider the function $\mathcal {S}(q_0,p_T)$ given by the extremal value of the constrained action functional $\mathfrak {S}$ over the family of curves $(q(\cdot ),p(\cdot ) , \lambda (\cdot ))$ satisfying the boundary conditions $q(0)=q_0$ and $p(T)=p_T$:

3.10 $$\begin{aligned} \mathcal {S}(q_0,p_T) = \mathop {\mathrm {ext}}\limits _{ \begin{array}{c} (q,p,\lambda )\in C^2([0,T],T^*\mathcal {Q} \times \Lambda ) \\ q(0)=q_0, \quad p(T)=p_T \end{array}}{\mathfrak {S} (q(\cdot ),p(\cdot ) , \lambda (\cdot )) }. \end{aligned}$$

The following theorem shows that $\mathcal {S}(q_0,p_T)$ is a generating function for the flow of the continuous constrained Hamilton’s equations:

#### Theorem 3.3

The exact time-*T* flow map of Hamilton’s equations $(q_0,p_0) \mapsto (q_T,p_T)$ is implicitly given by the following relations:

3.11 $$\begin{aligned} q_T = D_2 \mathcal {S} (q_0,p_T), \qquad p_0 = D_1 \mathcal {S} (q_0,p_T). \end{aligned}$$

In particular, $\mathcal {S} (q_0,p_T)$ is a Type II generating function that generates the exact flow of the constrained Hamilton’s Eq. (3.6).

#### Proof

See Appendix A.5. $$\square $$

In the Type III case, consider the function $\mathcal {S}(q_T,p_0)$ given by the extremal value of the constrained action functional $\mathfrak {S}$ over the family of curves $(q(\cdot ),p(\cdot ) , \lambda (\cdot ))$ satisfying the boundary conditions $q(T)=q_T$ and $p(0)=p_0$:

3.12 $$\begin{aligned} \mathcal {S}(q_T,p_0) = \mathop {\mathrm {ext}}\limits _{ \begin{array}{c} (q,p,\lambda )\in C^2([0,T],T^*\mathcal {Q} \times \Lambda ) \\ q(T)=q_T, \quad p(0)=p_0 \end{array}}{\mathfrak {S} (q(\cdot ),p(\cdot ) , \lambda (\cdot )) }. \end{aligned}$$

The following theorem shows that $\mathcal {S}(q_T,p_0)$ is a generating function for the flow of the continuous constrained Hamilton’s equations:

#### Theorem 3.4

The exact time-*T* flow map of Hamilton’s equations $(q_0,p_0) \mapsto (q_T,p_T)$ is implicitly given by the following relations:

3.13 $$\begin{aligned} q_0 = - D_2 \mathcal {S} (q_T,p_0), \qquad p_T = - D_1 \mathcal {S} (q_T,p_0). \end{aligned}$$

In particular, $\mathcal {S} (q_T,p_0)$ is a Type III generating function that generates the exact flow of the constrained Hamilton’s Eq. (3.8).

#### Proof

See Appendix A.6. $$\square $$

### Discrete Constrained Variational Hamiltonian Mechanics

Let us now extend the results of Sect. 3 from Leok and Zhang (2011) to introduce a discrete formulation of variational Hamiltonian mechanics which includes holonomic constraints. Suppose we are given a partition $0 = t_0< t_1< \ldots < t_N = T$ of the interval [0, *T*], and a discrete curve in $T^* \mathcal {Q} \times \Lambda$, denoted by $\{ (q_k,p_k, \lambda _k) \}_{k=0}^{N}$, such that $q_k \approx q(t_k)$, $p_k \approx p(t_k)$ and $\lambda _k \approx \lambda (t_k)$.

We formulate discrete constrained variational Hamiltonian mechanics in terms of the following discrete analogues of the constrained action functional $\mathfrak {S}$ given by Eq. (3.5):

3.14 $$\begin{aligned} \mathfrak {S}_d^+ \left( \{ (q_k,p_k, \lambda _k) \}_{k=0}^{N} \right)&\! =\! p_N q_N - \sum _{k=0}^{N-1}{\left[ p_{k+1} q_{k+1}\! -\! H_d^+(q_k,p_{k+1}) \!-\! \langle \lambda _k , \mathcal {C}(q_k) \rangle \right] }, \end{aligned}$$

3.15 $$\begin{aligned} \mathfrak {S}_d^- \left( \{ (q_k,p_k, \lambda _k) \}_{k=0}^{N} \right)&\!=\! -p_0 q_0 \!-\! \sum _{k=0}^{N-1}{\left[ \!-\! p_{k} q_{k}\! -\! H_d^-(q_{k+1},p_k)\! -\! \langle \lambda _{k+1} , \mathcal {C} (q_{k+1}) \rangle \right] }, \end{aligned}$$

where

3.16 $$\begin{aligned} H_d^+(q_k,p_{k+1})&\approx \mathop {\mathrm {ext}}\limits _{ \begin{array}{c} (q,p,\lambda )\in C^2([t_k,t_{k+1}],T^*\mathcal {Q} \times \Lambda ) \\ q(t_k)=q_k,\quad p(t_{k+1})=p_{k+1} \end{array}} p(t_{k+1}) q(t_{k+1}) \nonumber \\&\quad \quad \quad \quad \qquad \,\, - \int _{t_k}^{t_{k+1}}{\left[ p(t) \dot{q}(t) - H(q(t),p(t)) \right] dt} \end{aligned}$$

3.17 $$\begin{aligned} H_d^-(q_{k+1},p_k)&\approx \mathop {\mathrm {ext}}\limits _{ \begin{array}{c} (q,p,\lambda )\in C^2([t_k,t_{k+1}],T^*\mathcal {Q} \times \Lambda ) \\ q(t_{k+1})=q_{k+1}, \quad p(t_{k})=p_{k} \end{array}}-p(t_{k}) q(t_{k}) \nonumber \\&\qquad \qquad \qquad \quad - \int _{t_k}^{t_{k+1}}{\left[ p(t) \dot{q}(t) - H(q(t),p(t)) \right] dt} . \end{aligned}$$

We can now derive discrete analogues of Theorems 3.1 and 3.2 relating discrete variational principles to discrete constrained Hamilton’s equations, generalizing Lemma 3.1 from Leok and Zhang (2011):

#### Theorem 3.5

The Type II discrete Hamilton’s phase space variational principle

3.18 $$\begin{aligned} \delta \mathfrak {S}_d^{+} \left( \{ (q_k,p_k, \lambda _k) \}_{k=0}^{N} \right) = 0 \end{aligned}$$

is equivalent to the **discrete constrained right Hamilton’s equations**

3.19 $$\begin{aligned} q_{k+1} = D_2 H_d^+(q_{k},p_{k+1}), \quad p_k = D_1H_d^+(q_k,p_{k+1}) + \langle \lambda _k, \nabla \mathcal {C}(q_k) \rangle , \quad \mathcal {C}(q_k) =0,\nonumber \\ \end{aligned}$$

where $$H_d^+ (q_k,p_{k+1}) $$ is defined via Eq. (3.16).

#### Proof

See Appendix A.8. $$\square $$

#### Theorem 3.6

The Type III discrete Hamilton’s phase space variational principle

3.20 $$\begin{aligned} \delta \mathfrak {S}_d^{-} \left( \{ (q_k,p_k, \lambda _k) \}_{k=0}^{N} \right) = 0 \end{aligned}$$

is equivalent to the **discrete constrained left Hamilton’s equations**

3.21 $$\begin{aligned} q_{k}= & {} -D_2 H_d^-(q_{k+1},p_{k}), \quad p_{k+1} = -D_1H_d^-(q_{k+1},p_{k}) - \langle \lambda _{k+1}, \nabla \mathcal {C}(q_{k+1}) \rangle ,\nonumber \\&\quad \mathcal {C}(q_k) =0, \end{aligned}$$

where $$H_d^- (q_{k+1},p_{k}) $$ is defined via Eq. (3.17).

#### Proof

See Appendix A.9. $$\square $$

#### Remark 3.3

These discrete constrained Hamilton’s equations can be thought of as the discrete Hamilton’s equations generated by the augmented discrete Hamiltonians3.223.23This augmented Hamiltonian perspective together with the augmented Lagrangian perspective from Remark 2.2 imply that the constrained $\bar{H}_d^+$ variational integrator is equivalent to the constrained $\bar{L}_d^+$ variational integrator whenever the $H_d^+$ variational integrator is equivalent to the $L_d^+$ variational integrator (and similarly for the integrators generated by $\bar{H}_d^-$ and $\bar{L}_d^-$). Examples where this happens are presented in Schmitt et al. (2018) for Taylor variational integrators provided the Lagrangian is hyperregular, and in Leok and Zhang (2011) for generalized Galerkin variational integrators constructed using the same choices of basis functions and numerical quadrature formula provided the Hamiltonian is hyperregular.

## Error Analysis for Variational Integrators

### Unconstrained Error Analysis

Theorem 2.3.1 of Marsden and West (2001) states that if a discrete Lagrangian, $L_d:\mathcal {Q}\times \mathcal {Q}\rightarrow \mathbb {R}$, approximates the exact discrete Lagrangian $L_d^E:\mathcal {Q}\times \mathcal {Q}\rightarrow \mathbb {R}$ to order *r*, i.e.,

4.1 $$\begin{aligned} L_d(q_0, q_h)=L_d^E(q_0,q_h)+\mathcal {O}(h^{r+1}) , \end{aligned}$$

then the discrete Hamiltonian map $\tilde{F}_{L_d}:(q_k,p_k)\mapsto (q_{k+1},p_{k+1})$, viewed as a one-step method defined implicitly from the discrete Euler–Lagrange equations

4.2 $$\begin{aligned} D_1 L_d(q_k, q_{k+1}) +D_2 L_d(q_{k-1}, q_{k}) =0, \end{aligned}$$

or equivalently in terms of the implicit discrete Euler–Lagrange equations, which involve the corresponding discrete momenta via the discrete Legendre transforms,

4.3 $$\begin{aligned} p_k= -D_1L_d(q_k,q_{k+1}), \qquad p_{k+1}=D_2L_d(q_{k},q_{k+1}), \end{aligned}$$

has order of accuracy *r*.

Theorem 2.3.1 of Marsden and West (2001) has an analogue for Hamiltonian variational integrators. Theorem 2.2 in Schmitt and Leok (2017) states that if a discrete right Hamiltonian $H^+_d$ approximates the exact discrete right Hamiltonian $H_d^{+,E}$ to order *r*, i.e.,

4.4 $$\begin{aligned} H^+_d(q_0, p_h)=H_d^{+,E}(q_0,p_h)+\mathcal {O}(h^{r+1}), \end{aligned}$$

then the discrete right Hamiltonian map $\tilde{F}_{H^+_d}:(q_k,p_k)\mapsto (q_{k+1},p_{k+1})$, viewed as a one-step method defined implicitly by the discrete right Hamilton’s equations

4.5 $$\begin{aligned} p_k=D_1H_d^+(q_k,p_{k+1}), \qquad q_{k+1}=D_2H_d^+(q_k,p_{k+1}), \end{aligned}$$

is order *r* accurate. As mentioned in Schmitt and Leok (2017), the proof of Theorem 2.2 in Schmitt and Leok (2017) can easily be adjusted to prove an equivalent theorem for the discrete left Hamiltonian case, which states that if a discrete left Hamiltonian $H^-_d$ approximates the exact discrete left Hamiltonian $H_d^{-,E}$ to order *r*, i.e.,

4.6 $$\begin{aligned} H^-_d(q_1, p_0)=H_d^{-,E}(q_1,p_0)+\mathcal {O}(h^{r+1}), \end{aligned}$$

then the discrete left Hamiltonian map $\tilde{F}_{H^-_d}:(q_k,p_k)\mapsto (q_{k+1},p_{k+1})$, viewed as a one-step method defined implicitly by the discrete left Hamilton’s equations

4.7 $$\begin{aligned} p_{k+1}= - D_1H_d^-(q_{k+1},p_k), \qquad q_k = - D_2H_d^-(q_{k+1},p_k), \end{aligned}$$

is order *r* accurate. Many other properties of the integrator, such as momentum conservation properties of the method, can be determined by analyzing the associated discrete Lagrangian or Hamiltonian, as opposed to analyzing the integrator directly. We will exploit these error analysis results to derive analogous results for the constrained versions discussed in Sects. 2 and 3.

### Constrained Error Analysis

For the Lagrangian case, we can think of the Lagrange multipliers $\lambda$ as extra position coordinates and define an augmented Lagrangian $\bar{L}$ via

4.8 $$\begin{aligned} \bar{L}\left( (q,\lambda ),(\dot{q}, \dot{\lambda })\right) = L(q,\dot{q}) - \langle \lambda , \mathcal {C}(q) \rangle . \end{aligned}$$

A corresponding augmented discrete Lagrangian is given by

4.9 $$\begin{aligned} \bar{L}_d \left( (q_k,\lambda _k),(q_{k+1},\lambda _{k+1})\right) = L_d(q_k,q_{k+1}) - \langle \lambda _k , \mathcal {C}(q_k) \rangle , \end{aligned}$$

and the discrete Euler–Lagrange Eq. (4.2)

4.10 $$\begin{aligned} D_1 \bar{L}_d \left( (q_k,\lambda _k),(q_{k+1},\lambda _{k+1})\right) +D_2 \bar{L}_d \left( (q_{k-1},\lambda _{k-1}),(q_{k},\lambda _{k})\right) =0, \end{aligned}$$

yield the discrete constrained Euler–Lagrange equations

4.11 $$\begin{aligned}&D_1 L_d(q_k,q_{k+1}) + D_2 L_d(q_{k-1},q_{k}) = \langle \lambda _k ,\nabla \mathcal {C}(q_k) \rangle ,\nonumber \\&\qquad \mathcal {C}(q_k) =0, \end{aligned}$$

derived in Sect. 2.2. As a consequence, we can apply Theorem 2.3.1 of Marsden and West (2001) to the augmented Lagrangian (4.8) and obtain the following result:

#### Theorem 4.1

Suppose that for an exact discrete Lagrangian $L_d^E$ and a discrete Lagrangian $L_d$,

4.12 $$\begin{aligned}&L_d(q_0, q_h) - \langle \lambda _0 , \mathcal {C}(q_0) \rangle =L_d^E(q_0,q_h) - \int _{0}^{h}{\langle \lambda (t) , \mathcal {C}(q(t)) \rangle dt} +\mathcal {O}(h^{r+1}). \end{aligned}$$

Then, the discrete map $(q_k,p_k,\lambda _k)\mapsto (q_{k+1},p_{k+1},\lambda _{k+1})$, viewed as a one-step method defined implicitly by the discrete constrained Euler–Lagrange equations, has order of accuracy *r*.

For the Hamiltonian case, we can think of the Lagrange multipliers $\lambda$ as extra position coordinates and define conjugate momenta , which are constants of motion since the time-derivative of $\lambda$ does not appear anywhere, and are constrained to be zero. The augmented Hamiltonian $\bar{H}$, given by4.13yields the following augmented left and right discrete Hamiltonians4.144.15and the discrete left and right Hamilton’s equations4.164.17yield the discrete constrained left Hamilton’s equations

4.18 $$\begin{aligned}&q_k = - D_2 H_d^-(q_{k+1},p_{k}), \qquad p_{k+1} = - D_1H_d^-(q_{k+1},p_{k}) - \langle \lambda _{k+1} ,\nabla \mathcal {C}(q_{k+1}) \rangle ,\nonumber \\&\quad \mathcal {C}(q_k) =0, \end{aligned}$$

and the discrete constrained right Hamilton’s equations

4.19 $$\begin{aligned} q_{k+1} = D_2 H_d^+(q_{k},p_{k+1}), \quad p_k = D_1H_d^+(q_k,p_{k+1}) + \langle \lambda _k, \nabla \mathcal {C}(q_k) \rangle , \quad \mathcal {C}(q_{k}) =0,\nonumber \\ \end{aligned}$$

derived in Sect. 3. As a consequence, we can apply Theorem 2.2 in Schmitt and Leok (2017) and its Type III analogue to the augmented Hamiltonians and obtain the following results

#### Theorem 4.2

Suppose that given an exact discrete right Hamiltonian $H_d^{+,E}$ and a discrete right Hamiltonian $H_d^+$, we have

4.20 $$\begin{aligned} H^+_d(q_0, p_h) + \langle \lambda _0 , \mathcal {C}(q_0) \rangle =H_d^{+,E}(q_0,p_h)+ \int _{0}^{h}{\langle \lambda (t) , \mathcal {C}(q(t)) \rangle dt} +\mathcal {O}(h^{r+1}). \end{aligned}$$

Then, the discrete map $(q_k,p_k,\lambda _k)\mapsto (q_{k+1},p_{k+1},\lambda _{k+1})$, viewed as a one-step method defined implicitly by the discrete constrained right Hamilton’s equations, has order of accuracy *r*.

#### Theorem 4.3

Suppose that given an exact discrete left Hamiltonian $H_d^{-,E}$ and a discrete left Hamiltonian $H_d^-$, we have

4.21 $$\begin{aligned} H^-_d(q_h, p_0) + \langle \lambda _h , \mathcal {C}(q_h) \rangle =H_d^{-,E}(q_h,p_0)+ \int _{0}^{h}{\langle \lambda (t) , \mathcal {C}(q(t)) \rangle dt} +\mathcal {O}(h^{r+1}).\nonumber \\ \end{aligned}$$

Then, the discrete map $(q_k,p_k,\lambda _k)\mapsto (q_{k+1},p_{k+1},\lambda _{k+1})$, viewed as a one-step method defined implicitly by the discrete constrained left Hamilton’s equations, has order of accuracy *r*.

## Variational Riemannian Accelerated Optimization

### Riemannian Geometry

We first introduce the main notions from Riemannian geometry that will be used throughout this section (see Absil et al. (2008); Boumal (2020); Duruisseaux and Leok (2022a); Jost (2017); Lee (2018); Lang (1999) for more details).

#### Definition 5.1

Suppose we have a Riemannian manifold $$\mathcal {Q}$$ with Riemannian metric $$g(\cdot ,\cdot ) = \langle \cdot , \cdot \rangle $$, represented by the positive-definite symmetric matrix $$(g_{ij}) $$ in local coordinates. Then, we define the **musical isomorphism** $$g^{\flat }:T\mathcal {Q} \rightarrow T^*\mathcal {Q}$$ via

$$\begin{aligned} g^{\flat }(u)(v) = g_q(u,v) \quad \forall q\in \mathcal {Q} \text { and } \forall u,v\in T_q\mathcal {Q}, \end{aligned}$$

and its **inverse musical isomorphism** $g^{\sharp }:T^*\mathcal {Q} \rightarrow T\mathcal {Q}$. The Riemannian metric $g(\cdot ,\cdot ) = \langle \cdot , \cdot \rangle$ induces a **fiber metric** $g^*(\cdot ,\cdot ) = \langle \langle \cdot , \cdot \rangle \rangle$ on $T^* \mathcal {Q}$ via

$$\begin{aligned} \langle \langle u , v \rangle \rangle = \langle g^{\sharp }(u), g^{\sharp }(v) \rangle \quad \forall u,v \in T^* \mathcal {Q}, \end{aligned}$$

represented by the positive-definite symmetric matrix $$(g^{ij})$$ in local coordinates, which is the inverse of the Riemannian metric matrix $$(g_{ij}) $$.

#### Definition 5.2

Denoting the differential of *f* by *df*, the **Riemannian gradient** $\mathrm{grad}f(q) \in T_q \mathcal {Q}$ at a point $q\in \mathcal {Q}$ of a smooth function $f:\mathcal {Q} \rightarrow \mathbb {R}$ is the tangent vector at *q* such that

$$\begin{aligned} \langle \mathrm{grad}f(q) , u \rangle = df(q) u \qquad \forall u\in T_q \mathcal {Q}. \end{aligned}$$

This can also be expressed in terms of the inverse musical isomorphism, $\mathrm{grad}f(q)=g^\sharp (df(q))$.

#### Definition 5.3

A **geodesic** in a Riemannian manifold $\mathcal {Q}$ is a parametrized curve $\gamma : [0,1] \rightarrow \mathcal {Q}$ which is of minimal local length, and is a generalization of the notion of straight line from Euclidean spaces to Riemannian manifolds. The other generalization of straight lines involves curves having zero “acceleration" or constant “speed," which requires the introduction of an affine connection. These two generalizations are equivalent if the Riemannian manifold is endowed with the Levi–Civita connection. Given two points $q,\tilde{q} \in \mathcal {Q}$, a vector in $T_q \mathcal {Q}$ can be transported to $T_{\tilde{q}}\mathcal {Q}$ along a geodesic $\gamma$ by an operation $\Gamma _q^{\tilde{q}}:T_q \mathcal {Q}\rightarrow T_{\tilde{q}}\mathcal {Q}$ called the **parallel transport along** $\gamma$.

#### Definition 5.4

The **Riemannian Exponential map** $Exp _q:T_q \mathcal {Q} \rightarrow \mathcal {Q}$ at $q\in \mathcal {Q}$ is defined via

$$\begin{aligned} Exp _q(v) = \gamma _v(1), \end{aligned}$$

where $\gamma _v$ is the unique geodesic in $\mathcal {Q}$ such that $\gamma _v(0) = q$ and $\gamma _v'(0) = v$, for any $v\in T_q \mathcal {Q}$. $Exp _q$ is a diffeomorphism in some neighborhood $U \subset T_q\mathcal {Q}$ containing 0, so we can define its inverse map, the **Riemannian Logarithm map** $Log _p : Exp _q(U) \rightarrow T_q \mathcal {Q}$.

#### Definition 5.5

A **retraction** on a manifold $\mathcal {Q}$ is a smooth mapping $\mathcal {R}: T\mathcal {Q} \rightarrow \mathcal {Q}$ such that for any $q \in \mathcal {Q}$, the restriction $\mathcal {R}_q : T_q\mathcal {Q} \rightarrow \mathcal {Q}$ of $\mathcal {R}$ to $T_q\mathcal {Q}$ satisfies

- $\mathcal {R}_q(0_q) = q$, where $0_q$ denotes the zero element of $T_q\mathcal {Q}$,
- $T_{0_q}\mathcal {R}_q = \mathbb {I}_{T_q\mathcal {Q} }$ with the canonical identification $T_{0_q}T_{q}\mathcal {Q} \simeq T_{q}\mathcal {Q}$, where $T_{0_q}\mathcal {R}_q$ is the tangent map of $\mathcal {R}$ at $0_q \in T_{q}\mathcal {Q}$ and $\mathbb {I}_{T_q\mathcal {Q} }$ is the identity map on $T_{q}\mathcal {Q}$.

The Riemannian Exponential map is a natural example of a retraction on a Riemannian manifold.

#### Definition 5.6

A subset *A* of a Riemannian manifold $\mathcal {Q}$ is called **geodesically uniquely convex** if every two points of *A* are connected by a unique geodesic in *A*. A function $f:\mathcal {Q} \rightarrow \mathbb {R}$ is called **geodesically convex** if for any two points $q,\tilde{q} \in \mathcal {Q}$ and a geodesic $\gamma$ connecting them,

$$\begin{aligned} f(\gamma (t)) \le (1-t) f(q) +t f(\tilde{q}) \qquad \forall t\in [0,1]. \end{aligned}$$

Note that if *f* is a smooth geodesically convex function on a geodesically uniquely convex subset *A*,

$$\begin{aligned} f(q) - f(\tilde{q}) \ge \langle \mathrm{grad}f(\tilde{q}) , Log _{\tilde{q}}(q) \rangle \qquad \forall q,\tilde{q} \in A. \end{aligned}$$

A function $f:A\rightarrow \mathbb {R}$ is called **geodesically **$\alpha$-** weakly quasi-convex** ($\alpha$-WQC) with respect to $q \in \mathcal {Q}$ for some $\alpha \in (0,1]$ if

$$\begin{aligned} \alpha \left( f(q) - f(\tilde{q})\right) \ge \langle \mathrm{grad}f(\tilde{q}) , Log _{\tilde{q}}(q) \rangle \qquad \forall \tilde{q} \in A. \end{aligned}$$

Note that a local minimum of a geodesically convex or $\alpha$-WQC function is also a global minimum.

#### Definition 5.7

Given a Riemannian manifold $\mathcal {Q}$ with sectional curvature bounded below by $K_{\min }$, and an upper bound *D* for the diameter of the domain of consideration, define

5.1 $$\begin{aligned} \zeta = {\left\{ \begin{array}{ll} \sqrt{-K_{\min }} D \coth { (\sqrt{-K_{\min }} D) } &{} \quad \text {if } K_{\min } < 0 \\ 1 &{} \quad \text {if } K_{\min } \ge 0 \end{array}\right. } . \end{aligned}$$

Note that $\zeta \ge 1$ since $x \coth {x} \ge 1$ for all real values of *x*.

### Hamiltonian Approach

Our approach consists in integrating the Riemannian Bregman Hamiltonian systems derived in Duruisseaux and Leok (2022a) which evolve on the Riemannian manifold $\mathcal {Q}$, via discrete constrained variational Hamiltonian integrators which enforce the numerical solution to lie on the Riemannian manifold $\mathcal {Q}$. With $\zeta$ given by Eq. (5.1), we know from Duruisseaux and Leok (2022a) that if we let $\lambda = \zeta$ in the geodesically convex case, and $\lambda = \zeta / \alpha$ in the geodesically $\alpha$-weakly quasi-convex case, we obtain the Direct approach Riemannian *p*-Bregman Hamiltonian

5.2 $$\begin{aligned} \bar{\mathcal {H}}_{p}(\bar{Q},\bar{R}) = \frac{p}{2(Q^t)^{\lambda p +1}} \langle \langle R , R\rangle \rangle + Cp(Q^t)^{(\lambda +1)p-1} f(Q) + R^t, \end{aligned}$$

and the Adaptive approach Riemannian $p\rightarrow \mathring{p}$ Bregman Hamiltonian

5.3 $$\begin{aligned} \bar{\mathcal {H}}_{p \rightarrow \mathring{p}}(\bar{Q},\bar{R})= & {} \frac{p^2}{2\mathring{p} (Q^t)^{\lambda p +\mathring{p}/p}} \langle \langle R , R\rangle \rangle + \frac{Cp^2}{\mathring{p}}(Q^t)^{(\lambda +1)p-\mathring{p}/p} f(Q)\nonumber \\&\quad + \frac{p}{\mathring{p}} (Q^t)^{1-\mathring{p}/p} R^t. \end{aligned}$$

It is proven in Duruisseaux and Leok (2022a) that along the trajectories of the Riemannian *p*-Bregman dynamics, *f*(*Q*(*t*)) converges to its optimal value at a rate of $\mathcal {O}(1/t^p)$, under suitable assumptions on $\mathcal {Q}$.

#### Remark 5.1

In the vector space setting, these Riemannian Bregman Hamiltonians reduce to the direct and adaptive approach Bregman Hamiltonians derived in Duruisseaux et al. (2021) for convex functions:

5.4 $$\begin{aligned}&\bar{H}_{p}(\bar{q},\bar{r}) = \frac{p}{2(q^t)^{p+1}} \langle r , r \rangle + Cp(q^t)^{2p-1} f(q) + r^t , \end{aligned}$$

5.5 $$\begin{aligned}&\bar{H}_{p\rightarrow \mathring{p}}(\bar{q},\bar{r}) = \frac{p^2}{2\mathring{p}(q^t)^{p+\mathring{p}/p} } \langle r , r \rangle + \frac{Cp^2}{\mathring{p}} (q^t)^{2p-\mathring{p}/p} f(q) + \frac{p}{\mathring{p}} (q^t)^{1-\mathring{p}/p} r^t .\nonumber \\ \end{aligned}$$

### Some Optimization Problems on Riemannian Manifolds

#### Rayleigh Quotient Minimization on the Unit Sphere

An eigenvector *v* corresponding to the largest eigenvalue of a symmetric $n \times n$ matrix *A* maximizes the Rayleigh quotient $\frac{v^\top Av}{ v^\top v}$ over $\mathbb {R}^n$. Thus, a unit eigenvector corresponding to the largest eigenvalue of the matrix *A* is a minimizer of the function $f(v) = - v^\top Av$ over the unit sphere $\mathcal {Q} = \mathbb {S}^{n-1}$, which can be thought of as a Riemannian submanifold with constant positive curvature $K=1$ of $\mathbb {R}^{n}$ endowed with the Riemannian metric inherited from the Euclidean inner product $g_v(u,w) = u^\top w$. Solving the Rayleigh quotient optimization problem efficiently is challenging when the given symmetric matrix *A* is ill-conditioned and high-dimensional. Note that an efficient algorithm that solves the above minimization problem can also be used to find eigenvectors corresponding to the smallest eigenvalue of *A* by using the fact that the eigenvalues of *A* are the negative of the eigenvalues of $-A$.

#### Eigenvalue and Procrustes Problems on the Stiefel Manifold

When endowed with the Riemannian metric $g_X(A,B) = \text {Trace}(A^\top B)$, the **Stiefel manifold**

5.6 $$\begin{aligned} \text {St}(m,n) = \{X\in \mathbb {R}^{n\times m} | X^\top X= I_m \} \end{aligned}$$

is a Riemannian submanifold of $\mathbb {R}^{n\times m}$. The tangent space at any $X \in \text {St}(m,n)$ is given by $T_X \text {St}(m,n) = \{ Z\in \mathbb {R}^{n\times m} | X^\top Z+Z^\top X=0 \},$ and the orthogonal projection $P_X$ onto $T_X \text {St}(m,n)$ is given by $P_X Z = Z - \frac{1}{2} X(X^\top Z + Z^\top X).$ A retraction on $\text {St}(m,n)$ is given by $\mathcal {R}_X(\xi ) = \text {qf} (X+\xi ) ,$ where $\text {qf}(A)$ denotes the *Q* factor of the QR factorization of the matrix $A\in \mathbb {R}^{n\times m}$ as $A=QR$ where $Q\in \text {St}(m,n)$ and *R* is an upper triangular $n\times m$ matrix with strictly positive diagonal elements (Absil et al. 2008).

A generalized eigenvector problem consists of finding the *m* smallest eigenvalues of a $n\times n$ symmetric matrix *A* and corresponding eigenvectors. This problem can be formulated as a Riemannian optimization problem on the Stiefel manifold $\text {St}(m,n)$ via the Brockett cost function

5.7 $$\begin{aligned} f:\text {St}(m,n) \rightarrow \mathbb {R}, \quad X\mapsto f(X) = \text {Trace}(X^\top AXN), \end{aligned}$$

where $N = \text {diag}(\mu _1 , \ldots , \mu _m)$ for arbitrary $0 \le \mu _1 \le \ldots \le \mu _m$. The columns of a global minimizer of *f* are eigenvectors corresponding to the *m* smallest eigenvalues of *A* (see Absil et al. (2008)). If we define $\bar{f} : \mathbb {R}^{n\times m} \rightarrow \mathbb {R}$ via $X\mapsto \bar{f}(X) = \text {Trace}(X^\top AXN),$ then *f* is the restriction of $\bar{f}$ to $\text {St}(m,n)$ so

5.8 $$\begin{aligned} \text {grad}f(X) = P_X \text {grad}\bar{f}(X), \quad \text {where } \text { grad}\bar{f}(X) = 2AXN. \end{aligned}$$

The unbalanced orthogonal Procrustes problem consists of minimizing the function

5.9 $$\begin{aligned} f:\text {St}(m,n) \rightarrow \mathbb {R}, \quad X\mapsto f(X) = \Vert AX-B \Vert _F^2 , \end{aligned}$$

on the Stiefel manifold $\text {St}(m,n)$, for given matrices $A \in \mathbb {R}^{l\times n }$ and $B \in \mathbb {R}^{l\times m }$ with $l \ge n$ and $l>m$, where $\Vert \cdot \Vert _F$ is the Frobenius norm $\Vert X \Vert _F^2 = \text {Trace}(X^\top X)= \sum _{ij}{X_{ij}^2}$. If we define $\bar{f} : \mathbb {R}^{n\times m} \rightarrow \mathbb {R}$ via $X\mapsto \bar{f}(X) = \Vert AX-B \Vert _F^2,$ then *f* is the restriction of $\bar{f}$ to $\text {St}(m,n)$ so

5.10 $$\begin{aligned} \text {grad}f(X) = P_X \text {grad}\bar{f}(X), \quad \text { where } \text { grad}\bar{f}(X) = 2A^\top (AX-B). \end{aligned}$$

Note that the special case where $n=m$ is the balanced orthogonal Procrustes problem. In this case, $\text {St}(m,n) = O(n)$ so $\Vert AX \Vert _F^2 = \Vert A \Vert _F^2$ and minimizing the function $f(X) = \Vert AX-B \Vert _F^2$ is replaced by the problem of maximizing $\text {Trace}(X^\top A^\top B)$ over $X\in O(n)$. A solution is then given by $X^* = UV^\top$ where $B^\top A = U \Sigma V^\top$ is the Singular Value Decomposition of $B^\top A$ with square orthogonal matrices *U* and *V*, and the solution is unique provided $B^\top A$ is nonsingular (see Eldén and Park (1999); Golub and Van Loan (2013)).

### Numerical Methods

#### **H**amiltonian **T**aylor **V**ariational **I**ntegrators (HTVIs)

HTVIs were first introduced in Schmitt et al. (2018). A discrete approximate Hamiltonian is constructed by approximating the flow map and the trajectory associated with the boundary values using a Taylor method, and approximating the integral by a quadrature rule. The Hamiltonian Taylor variational integrator is then generated by the discrete Hamilton’s equations. More explicitly, Type II HTVIs are constructed as follows:

(i) Construct the *r*-order and $(r+1)$-order Taylor methods $\Psi _h^{(r)}$ and $\Psi _h^{(r+1)}$ approximating the exact time-*h* flow map $\Phi _h : T^*Q \rightarrow T^*Q$.
(ii) Approximate $p(0)=p_0$ by the solution $\tilde{p}_0$ of $p_1 = \pi _{T^*Q} \circ \Psi _h^{(r)}(q_0,\tilde{p}_0) ,$ where $\pi _{T^*Q}:(q,p)\mapsto p$.
(iii) Choose a quadrature rule of order *s* with weights and nodes given by $(b_i,c_i)$ for $i=1,...,m$ and generate approximations $(q_{c_i},p_{c_i}) \approx (q(c_i h),p(c_i h))$ via $(q_{c_i},p_{c_i}) = \Psi _{c_i h}^{(r)}(q_0,\tilde{p}_0).$
(iv) Approximate $q_1$ via $\tilde{q}_1 = \pi _{Q} \circ \Psi _h^{(r+1)}(q_0,\tilde{p}_0),$ where $\pi _{Q}:(q,p)\mapsto q$.
(v) Use the continuous Legendre transform to obtain $\dot{q}_{c_i} = \frac{\partial H}{\partial p_{c_i}}$.
(vi) Apply the quadrature rule to obtain the associated discrete right Hamiltonian $H_d^+(q_0,p_1) = p_1 \tilde{q}_1 - h \sum _{i=1}^{m}{b_i \left[ p_{c_i} \dot{q}_{c_i} - H(q_{c_i},p_{c_i}) \right] }.$
(vii) The variational integrator is then defined by the discrete right Hamilton’s equations.

Note that the following error analysis result concerning the order of accuracy of HTVIs was derived in Duruisseaux et al. (2021) (it can be extended to the constrained case via the strategy and results of Sect. 4.2):

##### Theorem 5.1

If the Hamiltonian *H* and its partial derivative $\frac{\partial H}{\partial p}$ are Lipschitz continuous in both variables, then $H_d^+(q_0,p_1)$ approximates $H_d^{+,E}(q_0,p_1)$ with at least order of accuracy $\min {(r+1,s)}$.

By Theorem 2.2 in Schmitt and Leok (2017), the associated discrete Hamiltonian map has the same order of accuracy.

In this paper, we will use the Direct approach and Adaptive approach $r=0$ Type II HTVIs constructed in Duruisseaux et al. (2021) based on the Direct and Adaptive discrete right Hamiltonians (respectively)

5.11 $$\begin{aligned} H_d^+(\bar{q}_0,\bar{r}_1;h)&= r_1^\top q_0 + r_1^t q_0^t + h \frac{p}{2(q_0^t)^{p+1}} r_1^\top r_1 + hCp(q_0^t)^{2p-1} f(q_0) + hr_1^t , \end{aligned}$$

5.12 $$\begin{aligned} H_d^+(\bar{q}_0,\bar{r}_1;h)&= r_1^\top q_0 + r_1^t q_0^t \nonumber \\&\quad \, + h \frac{p^2}{2\mathring{p} (q_0^t)^{p+\frac{\mathring{p}}{p}} } r_1^\top r_1 + hC \frac{p^2}{\mathring{p}} (q_0^t)^{2p-\frac{\mathring{p}}{p}} f(q_0) + h \frac{p}{\mathring{p}} (q_0^t)^{1-\frac{\mathring{p}}{p}} r_1^t . \end{aligned}$$

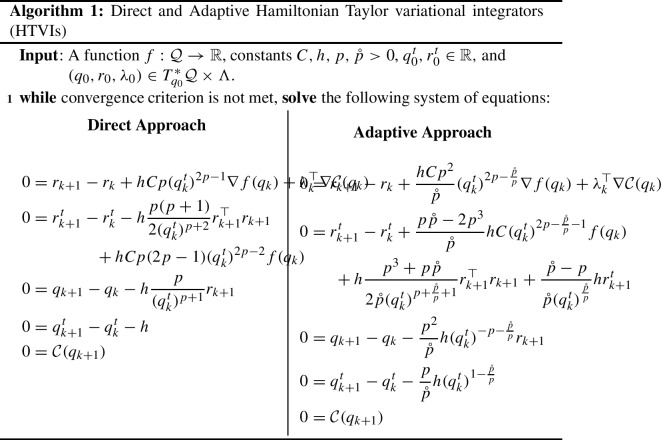


#### Euler–Lagrange Simple Discretization

In Duruisseaux and Leok (2022a), the *p*-Bregman Euler–Lagrange equations were rewritten as a first-order system of differential equations, for which a Riemannian version of a semi-implicit Euler scheme was applied to obtain the following algorithm: 
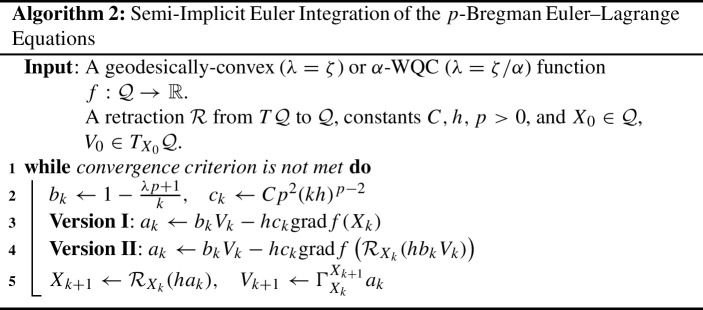


Version I of Algorithm 2 corresponds to the usual update for the semi-implicit Euler scheme, while Version II is inspired by the reformulation of Nesterov’s method from Sutskever et al. (2013) that uses a corrected gradient $\nabla f(X_k +h b_kV_k)$ instead of the traditional gradient $\nabla f(X_k)$.

#### **R**iemannian **G**radient **D**escent (RGD)

This is a generalization of Gradient Descent to the setting of Riemannian manifolds which involves the Riemannian gradient and a retraction. 
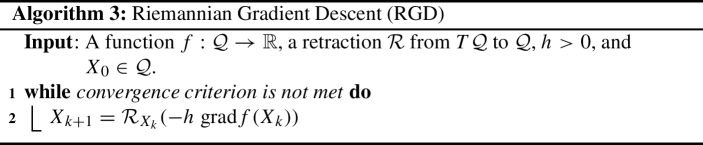


### Numerical Results

It is noted in Duruisseaux and Leok (2022a) that although higher values of *p* in Algorithm 2 result in provably faster rates of convergence, they also appear to be more prone to stability issues under numerical discretization, which can cause the numerical optimization algorithm to diverge. Numerical experiments in Duruisseaux et al. (2021) showed that in the normed vector space setting, geometric discretizations which respect the time-rescaling invariance and symplecticity of the Bregman Lagrangian and Hamiltonian flows were substantially less prone to these stability issues, and were therefore more robust, reliable, and computationally efficient. This was one of the motivations to develop time-adaptive Hamiltonian variational integrators for the Bregman Hamiltonians. Numerical experiments were conducted for the Rayleigh quotient minimization problem on $\mathbb {S}^{n-1}$, and for the generalized eigenvalue and Procrustes problems on the Stiefel manifold $\text {St}(m,n)$.

**Fig. 1:**
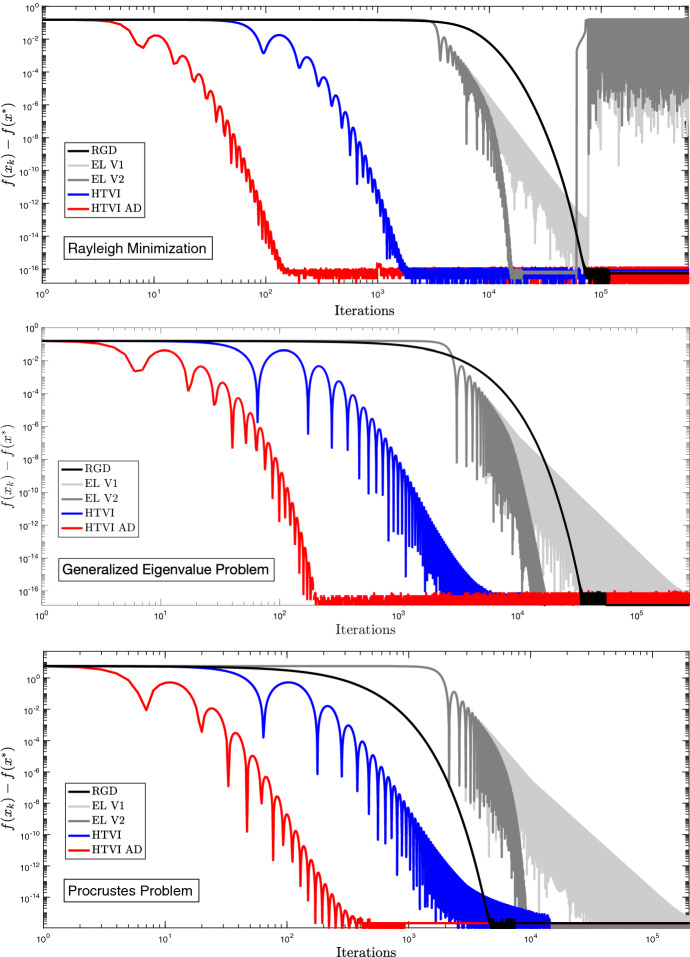
Comparison of the Direct and Adaptive (AD) Type II HTVIs with the Riemannian Gradient Descent (RGD) method and the Euler–Lagrange discretizations (EL V1 and EL V2) from Duruisseaux and Leok (2022a) with $p=6$ and the same timestep $h = 0.001$, for the Rayleigh quotient minimization problem on the unit sphere $\mathbb {S}^{n-1}$, and for the generalized eigenvalue and Procrustes problems on the Stiefel manifold $\text {St}(m,n)$

The results from Fig. 1 show how the Hamiltonian Taylor variational integrators compare to the Euler–Lagrange discretizations from Duruisseaux and Leok (2022a) and the standard Riemannian gradient descent. Note that for certain instances of the Procrustes problem with certain initial values, all the algorithms converged to a local minimizer, and not the global minimizer, of the objective function. We can observe from Fig. 1 that for the same value of the timestep *h*, the Adaptive Hamiltonian variational integrator clearly outperforms its Direct counterpart, Riemannian gradient descent and the Euler–Lagrange discretizations in terms of number of iterations required. Furthermore, unlike the Euler–Lagrange discretizations (Algorithm 2) and the Riemannian gradient descent (Algorithm 3), the HTVI methods (Algorithm 1) do not require the use of retractions or parallel transports. Note that the Rayleigh minimization results indicate that the Euler–Lagrange discretizations suffer from stability issues leading to a loss of convergence, as the polynomially growing unbounded coefficient $$C p^2 (kh)^{p-2} $$ is multiplied with $$\text {grad} f$$, so for this product to be bounded, the gradient has to decay to zero, but due to finite numerical precision, the gradient remains bounded away from zero, thereby causing the product to grow without bound. This issue can be resolved by adding a suitable upper bound to the coefficient $$C p^2 (kh)^{p-2} $$ in the updates, as can be seen both for the Euler–Lagrange discretizations and Hamiltonian variational integrators for the problems on $$\text {St}(m,n)$$.

However, the algorithms generated by these constrained Hamiltonian variational integrators are implicit, which can significantly increase the cost per iteration as the dimension of the problem becomes very large. In this case, it might be beneficial to consider other options using the unconstrained explicit Hamiltonian Taylor variational integrator, such as incorporating the constraints within the objective function as a penalty, although this might not constrain the solution trajectory to lie exactly on the manifold, or using projections if they can be computed efficiently and accurately for the Riemannian manifold of interest (Duruisseaux and Leok 2021). Further, note that the implementation of the Hamiltonian variational integrators needs a very careful tuning of the various parameters at play, which may be challenging and thus also motivates the development of different methods.

## Conclusion

Motivated by variational formulations of optimization problems on Riemannian manifolds, we first studied the relationship between the constrained Type I/II/III variational principles and the corresponding constrained Hamilton’s or Euler–Lagrange equations both in continuous and discrete time, and derived variational error analysis results for the maps defined implicitly by the resulting discrete constrained equations. We then exploited these discrete constrained variational integrators and the variational formulation of accelerated optimization on Riemannian manifolds from Duruisseaux and Leok (2022a) to numerically solve the generalized eigenvalue and Procrustes problems on $\mathbb {S}^{n-1}$ and $\text {St}(m,n)$.

The numerical experiments conducted in this paper corroborated the observation made for the vector space setting in Duruisseaux et al. (2021) that the adaptive Hamiltonian variational integrator is significantly more efficient than the direct Hamiltonian variational integrator, and that it can significantly outperform the Euler–Lagrange discretizations and Riemannian gradient descent, when its parameters are tuned carefully. Furthermore, it was noted that unlike the Euler–Lagrange discretizations and Riemannian gradient descent, the Hamiltonian algorithms did not require the use of retractions or parallel transports, which could be important when the problem considered lies on a Riemannian manifold for which it might not be possible to compute or approximate these objects efficiently.

We noted however that tuning the parameters of these discrete constrained variational integrators can be challenging, and also that the resulting algorithms are implicit, which may significantly increase the cost per iteration as the dimension of the problem becomes very large, in which case it might be beneficial to consider using the unconstrained explicit HTVIs with projections (Duruisseaux and Leok 2021) or by incorporating the constraints within the objective function as a penalty. Moreover, although the Whitney and Nash embedding theorems (Whitney 1944a, b; Nash 1956) imply that there is no loss of generality when studying Riemannian manifolds only as submanifolds of Euclidean spaces, there are limitations to the constrained integration strategy based on embeddings presented in this paper, and an approach intrinsically defined on Riemannian manifolds would be desirable. Indeed, the embedding approach usually leads to higher-dimensional computations, and requires an effective way of constructing the embedding or a natural way of writing down equations that constrain the problem and the numerical solutions to the Riemannian manifold. Furthermore, most results in Riemannian geometry or results concerning specific Riemannian manifolds are proven from an intrinsic perspective because the embedding approach tends to flood intrinsic geometric properties of the manifold with superfluous information coming from the additional dimensions of the Euclidean space. This motivates the development of intrinsic methods that would exploit the symmetries and geometric properties of the manifold and of the problem at hand.

Developing an intrinsic extension of Hamiltonian variational integrators to manifolds will require some additional work, since the current approach involves Type II/III generating functions $H_d^+(q_k, p_{k+1})$, $H_d^-(p_k, q_{k+1})$, which depend on the position at one boundary point, and the momentum at the other boundary point. However, this does not make intrinsic sense on a manifold, since one needs the base point in order to specify the corresponding cotangent space, and one should ideally consider a Hamiltonian variational integrator construction based on discrete Dirac mechanics (Leok and Ohsawa 2011), which would yield a generating function $E_d^+(q_k, q_{k+1}, p_{k+1})$, $E_d^-(q_k, p_k, q_{k+1})$, that depends on the position at both boundary points and the momentum at one of the boundary points. This approach can be viewed as a discretization of the generalized energy $E(q,v,p)=\langle p,v\rangle - L(q,v)$, in contrast to the Hamiltonian $H(q,p)=\mathop {\mathrm {ext}}\limits _{v}\langle p,v\rangle - L(q,v)=\left. \langle p,v\rangle - L(q,v)\right| _{p=\frac{\partial L}{\partial v}}$. On the other hand, the formulation of Lagrangian variational integrators presented in the introduction of Sect. 2 makes sense intrinsically on manifolds, and a framework for variable time-stepping in Lagrangian variational integration was introduced in our subsequent work (Duruisseaux and Leok 2022b) to design intrinsic time-adaptive Lagrangian variational integrators for accelerated optimization on Riemannian manifolds.

It would also be interesting to extend the proposed approach to the problem of optimization with nonintegrable constraints, which naturally leads to the question of whether vakonomic or nonholonomic mechanics is the appropriate description (Cortés et al. 2002). In the context of optimization with nonintegrable constraints, the relevant extension will likely involve vakonomic variational integrators (Benito and Martín de Diego 2005; Jiménez and Martín de Diego 2012). However, it would be interesting to relate the methods introduced in this paper to the existing work on variational integrators applied to optimal control problems (Junge et al. 2005; de León et al. 2007), and the discrete optimal control of nonholonomic dynamical systems would likely require a combination of the methods described here and nonholonomic integrators (Cortés and Martínez 2001; de León et al. 2004; Fedorov and Zenkov 2005; McLachlan and Perlmutter 2006).

## Data Availability

The datasets generated during and/or analyzed during the current study are available from the corresponding author on reasonable request.

## Appendix A. Proofs of Theorems for Constrained Variational Mechanics

### A.1. Proof of Theorem 2.1

#### Theorem A.1

Consider the constrained action functional $\mathfrak {S} : C^2([0,T],\mathcal {Q} \times \Lambda ) \rightarrow \mathbb {R}$ given by

A.1 $$\begin{aligned} \mathfrak {S} (q(\cdot ), \lambda (\cdot )) = \int _{0}^{T}{ \left[ L(q(t),\dot{q}(t)) - \langle \lambda (t) , \mathcal {C}(q(t)) \rangle \right] dt}. \end{aligned}$$

The condition that $\mathfrak {S} (q(\cdot ), \lambda (\cdot ))$ is stationary with respect to the boundary conditions $\delta q(0) = 0$ and $\delta q(T) =0$ is equivalent to $(q(\cdot ),\lambda (\cdot ))$ satisfying the constrained Euler–Lagrange equations

A.2 $$\begin{aligned} \frac{\partial L}{\partial q} - \frac{d}{dt} \frac{\partial L}{\partial \dot{q}} = \langle \lambda , \nabla \mathcal {C} (q) \rangle , \qquad \mathcal {C}(q) =0 . \end{aligned}$$

#### Proof

Computing the variation of $\mathfrak {S}$ yields

$$\begin{aligned} \delta \mathfrak {S}&= \int _{0}^{T}{ \left[ \frac{\partial L}{\partial q}(q(t),\dot{q}(t)) \delta q(t) + \frac{\partial L}{\partial \dot{q}}(q(t),\dot{q}(t)) \delta \dot{q}(t)\right] dt}\\&\quad - \int _{0}^{T}{ \left[ \langle \lambda (t) , \nabla \mathcal {C} (q(t)) \delta q(t) \rangle + \langle \delta \lambda (t) , \mathcal {C}(q(t)) \rangle \right] dt } . \end{aligned}$$

Using integration by parts and the boundary conditions $\delta q(0) = 0$ and $\delta q (T) = 0,$ we get

$$\begin{aligned} \delta \mathfrak {S}&=\int _{0}^{T}{ \left[ \frac{\partial L}{\partial q}(q(t),\dot{q}(t)) - \frac{d}{dt} \frac{\partial L}{\partial \dot{q}}(q(t),\dot{q}(t)) - \langle \lambda (t) , \nabla \mathcal {C} (q(t)) \rangle \right] \delta q(t) dt } \\&\quad - \int _{0}^{T}{ \langle \delta \lambda (t) , \mathcal {C}(q(t)) \rangle dt } . \end{aligned}$$

Now, if $\delta \mathfrak {S} = 0$, then the fundamental theorem of the calculus of variations (Arnol’d 1989) yields the constrained Euler–Lagrange Eq. (A.2). Conversely, if $(q,\lambda )$ satisfies the constrained Euler–Lagrange Eq. (A.2), then the integrand vanishes and $\delta \mathfrak {S} = 0$. $\square$

### A.2. Proof of Theorem 3.1

#### Theorem A.2

Consider the constrained action functional $\mathfrak {S} : C^2([0,T],T^*\mathcal {Q} \times \Lambda ) \rightarrow \mathbb {R}$ given by

A.3 $$\begin{aligned} \mathfrak {S} (q(\cdot ),p(\cdot ), \lambda (\cdot ))= & {} p(T)q(T) \nonumber \\- & {} \int _{0}^{T}{ \left[ p(t) \dot{q}(t) - H(q(t),p(t)) - \langle \lambda (t) , \mathcal {C}(q(t)) \rangle \right] dt}.\nonumber \\ \end{aligned}$$

The condition that $\mathfrak {S} (q(\cdot ),p(\cdot ), \lambda (\cdot ))$ is stationary with respect to the boundary conditions $\delta q(0) = 0$ and $\delta p(T) =0$ is equivalent to $(q(\cdot ),p(\cdot ),\lambda (\cdot ))$ satisfying Hamilton’s canonical constrained equations

A.4 $$\begin{aligned} \dot{q} = \frac{\partial H}{\partial p} (q,p) , \qquad \dot{p} = -\frac{\partial H}{\partial q} (q,p) - \langle \lambda , \nabla \mathcal {C} (q) \rangle , \qquad \mathcal {C}(q) = 0 . \end{aligned}$$

#### Proof

Computing the variation of $\mathfrak {S}$ yields

A.5 $$\begin{aligned} \delta \mathfrak {S}&= q(T) \delta p(T) + p(T) \delta q (T) + \int _{0}^{T}{ \left[ \langle \lambda (t) , \nabla \mathcal {C} (q(t)) \delta q(t) \rangle + \langle \delta \lambda (t) , \mathcal {C}(q(t)) \rangle \right] dt } \nonumber \\&\quad - \int _{0}^{T}{ \left[ \dot{q} (t) \delta p(t) + p(t) \delta \dot{q}(t) - \frac{\partial H}{\partial q}(q(t),p(t)) \delta q(t) - \frac{\partial H}{\partial p}(q(t),p(t)) \delta p(t) \right] dt }. \end{aligned}$$

Using integration by parts and the boundary conditions $\delta q(0) = 0$ and $\delta p (T) = 0,$ we get

$$\begin{aligned} \delta \mathfrak {S}&= q(T) \delta p(T) + p(T) \delta q(T) - p(T) \delta q(T) + p(0) \delta q(0) +\int _{0}^{T}{ \langle \delta \lambda (t) , \mathcal {C}(q(t)) \rangle dt } \\&\quad + \int _{0}^{T}{ \left[ \dot{p}(t) + \frac{\partial H}{\partial q}(q(t),p(t)) + \langle \lambda (t) , \nabla \mathcal {C} (q(t)) \rangle \right] \delta q(t) dt } + \int _{0}^{T}{ \left[ \frac{\partial H}{\partial p}(q(t),p(t)) -\dot{q} (t) \right] \delta p(t) dt } \\&= \int _{0}^{T}{ \left[ \dot{p}(t) + \frac{\partial H}{\partial q}(q(t),p(t)) + \langle \lambda (t) , \nabla \mathcal {C} (q(t)) \rangle \right] \delta q(t) dt } + \int _{0}^{T}{ \left[ \frac{\partial H}{\partial p}(q(t),p(t)) -\dot{q} (t) \right] \delta p(t) dt } \\&\quad + \int _{0}^{T}{ \langle \delta \lambda (t) , \mathcal {C}(q(t)) \rangle dt } . \end{aligned}$$

Now, if $\delta \mathfrak {S} = 0$, then the fundamental theorem of the calculus of variations (Arnol’d 1989) yields Hamilton’s constrained Eq. (A.4). Conversely, if $(q,p,\lambda )$ satisfies Hamilton’s constrained Eq. (A.4), then the integrand vanishes and $\delta \mathfrak {S} = 0$. $\square$

### A.3. Proof of Theorem 3.2

#### Theorem A.3

Consider the constrained action functional $\mathfrak {S} : C^2([0,T],T^*\mathcal {Q} \times \Lambda ) \rightarrow \mathbb {R}$ given by

A.6 $$\begin{aligned} \mathfrak {S} (q(\cdot ),p(\cdot ), \lambda (\cdot ))= & {} - p(0)q(0) \nonumber \\- & {} \int _{0}^{T}{ \left[ p(t) \dot{q}(t) - H(q(t),p(t)) - \langle \lambda (t) , \mathcal {C}(q(t)) \rangle \right] dt}.\nonumber \\ \end{aligned}$$

The condition that $\mathfrak {S} (q(\cdot ),p(\cdot ), \lambda (\cdot ))$ is stationary with respect to the boundary conditions $\delta q(T) = 0$ and $\delta p(0) =0$ is equivalent to $(q(\cdot ),p(\cdot ),\lambda (\cdot ))$ satisfying Hamilton’s canonical constrained equations

A.7 $$\begin{aligned} \dot{q} = \frac{\partial H}{\partial p} (q,p) , \qquad \dot{p} = -\frac{\partial H}{\partial q} (q,p) - \langle \lambda , \nabla \mathcal {C} (q) \rangle , \qquad \mathcal {C}(q) = 0 . \end{aligned}$$

#### Proof

The proof is almost identical to that of Theorem 3.1. We compute the variation of $\mathfrak {S}$ as before and get Eq. (A.5) except that the term $\left( q(T) \delta p(T) + p(T) \delta q (T) \right)$ is replaced by $\left( - q(0) \delta p(0) - p(0) \delta q (0)\right)$. As before, integration by parts and the boundary conditions $\delta q(T) = 0$ and $\delta p(0) =0$ yield

$$\begin{aligned} \delta \mathfrak {S}&= \int _{0}^{T}{ \left[ \dot{p}(t) + \frac{\partial H}{\partial q}(q(t),p(t)) + \langle \lambda (t) , \nabla \mathcal {C} (q(t)) \rangle \right] \delta q(t) dt } \\&\quad + \int _{0}^{T}{ \left[ \frac{\partial H}{\partial p}(q(t),p(t)) -\dot{q} (t) \right] \delta p(t) dt } \\&\quad + \int _{0}^{T}{ \langle \delta \lambda (t) , \mathcal {C}(q(t)) \rangle dt } . \end{aligned}$$

Then, if $\delta \mathfrak {S} = 0$, then the fundamental theorem of the calculus of variations (Arnol’d 1989) yields Hamilton’s constrained Eq. (A.7). Conversely, if $(q,p,\lambda )$ satisfies Hamilton’s constrained Eq. (A.7), then the integrand vanishes and $\delta \mathfrak {S} = 0$. $\square$

### A.4. Proof of Theorem 2.2

#### Theorem A.4

The exact time-*T* flow map of Hamilton’s equations $(q_0,p_0) \mapsto (q_T,p_T)$ is implicitly given by the following relations:

A.8 $$\begin{aligned} D_1 \mathcal {S} (q_0,q_T) = - \frac{\partial L}{ \partial \dot{q}}(q_0,\dot{q}(0)), \qquad D_2 \mathcal {S} (q_0,q_T) = \frac{\partial L}{ \partial \dot{q}}(q_T,\dot{q}(T)) . \end{aligned}$$

Thus, $\mathcal {S} (q_0,q_T)$ is a Type I generating function that generates the exact flow of the constrained Euler–Lagrange Eq. (2.6).

#### Proof

Using integration by parts and simplifying gives

$$\begin{aligned} \frac{ \partial \mathcal {S}}{ \partial q_0 }(q_0,q_T)&= \int _{0}^{T}{ \left[ \frac{\partial q(t)}{\partial q_0} \frac{\partial L}{\partial q}(q(t),\dot{q}(t)) + \frac{\partial \dot{q}(t)}{\partial q_0} \frac{\partial L}{\partial \dot{q}}(q(t),\dot{q}(t)) \right] dt } \\&\quad - \int _{0}^{T}{ \left[ \langle \lambda (t) , \frac{\partial q(t)}{\partial q_0} \nabla \mathcal {C} (q(t)) \rangle + \langle \frac{\partial \lambda (t) }{\partial q_0} , \mathcal {C}(q(t)) \rangle \right] dt} \\&= \int _{0}^{T}{ \frac{\partial q(t)}{\partial q_0} \left( \frac{\partial L}{\partial q}(q(t),\dot{q}(t)) - \frac{d}{dt} \frac{\partial L}{\partial \dot{q}}(q(t),\dot{q}(t)) - \langle \lambda (t) , \nabla \mathcal {C} (q(t)) \rangle \right) dt} \\&\quad - \int _{0}^{T}{ \langle \frac{\partial \lambda (t) }{\partial q_0} , \mathcal {C}(q(t)) \rangle dt} - \frac{\partial L}{ \partial \dot{q}}(q(0),\dot{q}(0)) , \\ \frac{ \partial \mathcal {S}}{ \partial q_T }(q_0,q_T)&= \int _{0}^{T}{ \left[ \frac{\partial q(t)}{\partial q_T} \frac{\partial L}{\partial q}(q(t),\dot{q}(t)) + \frac{\partial \dot{q}(t)}{\partial q_T} \frac{\partial L}{\partial \dot{q}}(q(t),\dot{q}(t)) \right] dt } \\&\quad - \int _{0}^{T}{ \left[ \langle \lambda (t) , \frac{\partial q(t)}{\partial q_T} \nabla \mathcal {C} (q(t)) \rangle + \langle \frac{\partial \lambda (t) }{\partial q_T} , \mathcal {C}(q(t)) \rangle \right] dt} \\&= \int _{0}^{T}{ \frac{\partial q(t)}{\partial q_T} \left( \frac{\partial L}{\partial q}(q(t),\dot{q}(t)) - \frac{d}{dt} \frac{\partial L}{\partial \dot{q}}(q(t),\dot{q}(t)) - \langle \lambda (t) , \nabla \mathcal {C} (q(t)) \rangle \right) dt} \\&\quad - \int _{0}^{T}{ \langle \frac{\partial \lambda (t) }{\partial q_T} , \mathcal {C}(q(t)) \rangle dt} + \frac{\partial L}{ \partial \dot{q}}(q(T),\dot{q}(T)). \end{aligned}$$

By Theorem 2.1, the extremum of the action functional $\mathfrak {S}$ is achieved when $(q,\lambda )$ satisfies the constrained Euler–Lagrange Eq. (2.6), so we get $D_1 \mathcal {S} (q_0,q_T) = - \frac{\partial L}{ \partial \dot{q}}(q_0,\dot{q}(0))$ and $D_2 \mathcal {S} (q_0,q_T) = \frac{\partial L}{ \partial \dot{q}}(q_T,\dot{q}(T))$. $\square$

### A.5. Proof of Theorem 3.3

#### Theorem A.5

The exact time-*T* flow map of Hamilton’s equations $(q_0,p_0) \mapsto (q_T,p_T)$ is implicitly given by the following relations:

A.9 $$\begin{aligned} q_T = D_2 \mathcal {S} (q_0,p_T), \qquad p_0 = D_1 \mathcal {S} (q_0,p_T). \end{aligned}$$

In particular, $\mathcal {S} (q_0,p_T)$ is a Type II generating function that generates the exact flow of Hamilton’s constrained Eq. (3.6).

#### Proof

Using integration by parts and simplifying gives

$$\begin{aligned} \frac{ \partial \mathcal {S}}{ \partial q_0 }(q_0,p_T)&= \frac{\partial q_T}{\partial q_0} p_T + \int _{0}^{T}{ \left[ \langle \lambda (t) , \frac{\partial q(t)}{\partial q_0} \nabla \mathcal {C} (q(t)) \rangle + \langle \frac{\partial \lambda (t) }{\partial q_0} , \mathcal {C}(q(t)) \rangle \right] dt} \\&\quad - \int _{0}^{T}{ \left[ \frac{\partial p(t)}{\partial q_0} \dot{q}(t) + \frac{\partial \dot{q}(t)}{\partial q_0} p(t) - \frac{\partial q(t)}{\partial q_0} \frac{\partial H}{\partial q}(q(t),p(t)) - \frac{\partial p(t)}{\partial q_0} \frac{\partial H}{\partial p}(q(t),p(t)) \right] dt } \\&= p_0 + \int _{0}^{T}{ \frac{\partial q(t)}{\partial q_0} \left( \dot{p}(t) + \frac{\partial H}{\partial q}(q(t),p(t)) + \langle \lambda (t) , \nabla \mathcal {C} (q(t)) \rangle \right) dt} \\&\quad - \int _{0}^{T}{ \frac{\partial p(t)}{\partial q_0} \left( \dot{q}(t) - \frac{\partial H}{\partial p}(q(t),p(t)) \right) dt} + \int _{0}^{T}{ \langle \frac{\partial \lambda (t) }{\partial q_0} , \mathcal {C}(q(t)) \rangle dt} , \\ \frac{ \partial \mathcal {S}}{ \partial p_T }(q_0,p_T)&= q_T + \frac{\partial q_T}{\partial p_T} p_T + \int _{0}^{T}{ \left[ \langle \lambda (t) , \frac{\partial q(t)}{\partial p_T} \nabla \mathcal {C} (q(t)) \rangle + \langle \frac{\partial \lambda (t) }{\partial p_T} , \mathcal {C}(q(t)) \rangle \right] dt} \\&\quad - \int _{0}^{T}{ \left[ \frac{\partial p(t)}{\partial p_T} \dot{q}(t) + \frac{\partial \dot{q}(t)}{\partial p_T} p(t) - \frac{\partial q(t)}{\partial p_T} \frac{\partial H}{\partial q}(q(t),p(t)) - \frac{\partial p(t)}{\partial p_T} \frac{\partial H}{\partial p}(q(t),p(t)) \right] dt } \\&= q_T + \int _{0}^{T}{ \frac{\partial q(t)}{\partial p_T} \left( \dot{p}(t) + \frac{\partial H}{\partial q}(q(t),p(t)) + \langle \lambda (t) , \nabla \mathcal {C} (q(t)) \rangle \right) dt} \\&\quad - \int _{0}^{T}{ \frac{\partial p(t)}{\partial p_T} \left( \dot{q}(t) - \frac{\partial H}{\partial p}(q(t),p(t)) \right) dt} + \int _{0}^{T}{ \langle \frac{\partial \lambda (t) }{\partial p_T} , \mathcal {C}(q(t)) \rangle dt} . \end{aligned}$$

By Theorem 3.1, the extremum of the action functional $\mathfrak {S}$ is achieved when the curve $(q,p,\lambda )$ satisfies Hamilton’s constrained Eq. (3.6), so the integrands vanish, and thus $p_0 = \frac{\partial \mathcal {S} }{ \partial q_0} (q_0,p_T) = D_1 \mathcal {S} (q_0,p_T)$ and $q_T = \frac{\partial \mathcal {S} }{ \partial p_T} (q_0,p_T) = D_2 \mathcal {S} (q_0,p_T)$. $\square$

### A.6. Proof of Theorem 3.4

#### Theorem A.6

The exact time-*T* flow map of Hamilton’s equations $(q_0,p_0) \mapsto (q_T,p_T)$ is implicitly given by the following relations:

A.10 $$\begin{aligned} q_0 = - D_2 \mathcal {S} (q_T,p_0), \qquad p_T = - D_1 \mathcal {S} (q_T,p_0). \end{aligned}$$

In particular, $\mathcal {S} (q_T,p_0)$ is a Type III generating function that generates the exact flow of Hamilton’s constrained Eq. (3.8).

#### Proof

Integrating by parts and simplifying yields

$$\begin{aligned} \frac{ \partial \mathcal {S}}{ \partial q_T }(q_T,p_0)&= - \frac{\partial q_0}{\partial q_T} p_0 + \int _{0}^{T}{ \left[ \langle \lambda (t) , \frac{\partial q(t)}{\partial q_T} \nabla \mathcal {C} (q(t)) \rangle + \langle \frac{\partial \lambda (t) }{\partial q_T} , \mathcal {C}(q(t)) \rangle \right] dt} \\&\quad - \int _{0}^{T}{ \left[ \frac{\partial p(t)}{\partial q_T} \dot{q}(t) + \frac{\partial \dot{q}(t)}{\partial q_T} p(t) - \frac{\partial q(t)}{\partial q_T} \frac{\partial H}{\partial q}(q(t),p(t)) - \frac{\partial p(t)}{\partial q_T} \frac{\partial H}{\partial p}(q(t),p(t)) \right] dt } \\&= - p_T + \int _{0}^{T}{ \frac{\partial q(t)}{\partial q_T} \left( \dot{p}(t) + \frac{\partial H}{\partial q}(q(t),p(t)) + \langle \lambda (t) , \nabla \mathcal {C} (q(t)) \rangle \right) dt} \\&\quad - \int _{0}^{T}{ \frac{\partial p(t)}{\partial q_T} \left( \dot{q}(t) - \frac{\partial H}{\partial p}(q(t),p(t)) \right) dt} + \int _{0}^{T}{ \langle \frac{\partial \lambda (t) }{\partial q_T} , \mathcal {C}(q(t)) \rangle dt} , \\ \frac{ \partial \mathcal {S}}{ \partial p_0 }(q_T,p_0)&= - q_0 - \frac{\partial q_0}{\partial p_0} p_0 +\int _{0}^{T}{ \left[ \langle \lambda (t) , \frac{\partial q(t)}{\partial p_0} \nabla \mathcal {C} (q(t)) \rangle + \langle \frac{\partial \lambda (t) }{\partial p_0} , \mathcal {C}(q(t)) \rangle \right] dt}\\&\quad - \int _{0}^{T}{ \left[ \frac{\partial p(t)}{\partial p_0} \dot{q}(t) + \frac{\partial \dot{q}(t)}{\partial p_0} p(t) - \frac{\partial q(t)}{\partial p_0} \frac{\partial H}{\partial q}(q(t),p(t)) - \frac{\partial p(t)}{\partial p_0} \frac{\partial H}{\partial p}(q(t),p(t)) \right] dt } \\&= -q_0 + \int _{0}^{T}{ \frac{\partial q(t)}{\partial p_0} \left( \dot{p}(t) + \frac{\partial H}{\partial q}(q(t),p(t)) + \langle \lambda (t) , \nabla \mathcal {C} (q(t)) \rangle \right) dt} \\&\quad - \int _{0}^{T}{ \frac{\partial p(t)}{\partial p_0} \left( \dot{q}(t) - \frac{\partial H}{\partial p}(q(t),p(t)) \right) dt} + \int _{0}^{T}{ \langle \frac{\partial \lambda (t) }{\partial p_0} , \mathcal {C}(q(t)) \rangle dt} . \end{aligned}$$

By Theorem 3.2, the extremum of the action $\mathfrak {S}$ is achieved when the curve $(q,p,\lambda )$ satisfies Hamilton’s constrained Eq. (3.8), so the integrands vanish, and thus $p_T = - \frac{\partial \mathcal {S} }{ \partial q_T} (q_T,p_0) = - D_1 \mathcal {S} (q_T,p_0)$ and $q_0 = - \frac{\partial \mathcal {S} }{ \partial p_0} (q_T,p_0) = - D_2 \mathcal {S} (q_T,p_0)$. $\square$

### A.7. Proof of Theorem 2.3

#### Theorem A.7

The Type I discrete Hamilton’s variational principles

A.11 $$\begin{aligned} \delta \mathfrak {S}_d^{\pm } \left( \{ (q_k, \lambda _k) \}_{k=0}^{N} \right) = 0 \end{aligned}$$

are equivalent to the discrete constrained Euler–Lagrange equations

A.12 $$\begin{aligned} D_1 L_d(q_k,q_{k+1}) + D_2 L_d(q_{k-1},q_{k}) = \langle \lambda _k ,\nabla \mathcal {C}(q_k) \rangle , \quad \mathcal {C}(q_{k}) =0, \end{aligned}$$

where $$L_d(q_{k},q_{k+1}) $$ is defined via Eq. (2.11).

#### Proof

Using the fact that $\delta q_0 =0$ and $\delta q_N =0$, we have

$$\begin{aligned} \delta \mathfrak {S}_d^-&= \delta \left( \sum _{k=0}^{N-1}{\left[ L_d(q_k,q_{k+1}) - \langle \lambda _k, \mathcal {C}(q_k) \rangle \right] } \right) \\&= \sum _{k=0}^{N-1}{\left[ D_1 L_d(q_k,q_{k+1}) \delta q_k + D_2 L_d(q_k,q_{k+1}) \delta q_{k+1} \right] }\\&\quad - \sum _{k=0}^{N-1}{\left( \langle \lambda _k ,\nabla \mathcal {C}(q_k) \delta q_k \rangle + \langle \delta \lambda _k , \mathcal {C}(q_k) \rangle \right) } \\&= \sum _{k=1}^{N-1}{\left[ D_1 L_d(q_k,q_{k+1}) + D_2 L_d(q_{k-1},q_{k}) - \langle \lambda _k ,\nabla \mathcal {C}(q_k) \rangle \right] \delta q_{k} } \\&\quad - \sum _{k=0}^{N-1}{\langle \delta \lambda _k , \mathcal {C}(q_k) \rangle }, \\ \delta \mathfrak {S}_d^+&= \delta \left( \sum _{k=0}^{N-1}{\left[ L_d(q_k,q_{k+1}) - \langle \lambda _{k+1}, \mathcal {C}(q_{k+1}) \rangle \right] } \right) \\&= \sum _{k=0}^{N-1}{\left[ D_1 L_d(q_k,q_{k+1}) \delta q_k + D_2 L_d(q_k,q_{k+1}) \delta q_{k+1} \right] } \\&\quad - \sum _{k=0}^{N-1}{\left( \langle \lambda _{k+1} ,\nabla \mathcal {C}(q_{k+1}) \delta q_{k+1} \rangle + \langle \delta \lambda _{k+1} , \mathcal {C}(q_{k+1}) \rangle \right) } \\&= \sum _{k=1}^{N-1}{\left[ D_1 L_d(q_k,q_{k+1}) + D_2 L_d(q_{k-1},q_{k}) - \langle \lambda _k ,\nabla \mathcal {C}(q_k) \rangle \right] \delta q_{k} }\\&\quad - \sum _{k=0}^{N-1}{\langle \delta \lambda _{k+1} , \mathcal {C}(q_{k+1}) \rangle }. \end{aligned}$$

If the discrete constrained Euler–Lagrange Eq. (A.12) are satisfied, then each term vanishes and $\delta \mathfrak {S}_d^{\pm } = 0$. Conversely, if $\delta \mathfrak {S}_d^{\pm } = 0$, then a discrete fundamental theorem of the calculus of variations yields the discrete constrained Euler–Lagrange Eq. (A.12). $\square$

### A.8. Proof of Theorem 3.5

#### Theorem A.8

The Type II discrete Hamilton’s phase space variational principle

A.13 $$\begin{aligned} \delta \mathfrak {S}_d^{+} \left( \{ (q_k,p_k, \lambda _k) \}_{k=0}^{N} \right) = 0 \end{aligned}$$

is equivalent to the discrete constrained right Hamilton’s equations

A.14 $$\begin{aligned} q_{k+1} = D_2 H_d^+(q_{k},p_{k+1}), \quad p_k = D_1H_d^+(q_k,p_{k+1}) + \langle \lambda _k, \nabla \mathcal {C}(q_k) \rangle , \quad \mathcal {C}(q_k) =0,\nonumber \\ \end{aligned}$$

where $$H_d^+ (q_k,p_{k+1}) $$ is defined via Eq. (3.16).

#### Proof

Using the fact that $\delta q_0 =0$ and $\delta p_N =0$ since $(q_0,p_N)$ is fixed, we obtain the following expression for the variations of $\mathfrak {S}_d^{+}$:

$$\begin{aligned} \delta \mathfrak {S}_d^+&= \delta \left( p_N q_N - \sum _{k=0}^{N-1}{\left[ p_{k+1} q_{k+1} - H_d^+(q_k,p_{k+1}) - \langle \lambda _k, \mathcal {C}(q_k) \rangle \right] } \right) \\&= \delta \left( -\sum _{k=0}^{N-2}{p_{k+1}q_{k+1}} + \sum _{k=0}^{N-1}{\left[ H_d^+(q_k,p_{k+1}) + \langle \lambda _k, \mathcal {C}(q_k) \rangle \right] } \right) \\&= -\sum _{k=0}^{N-2}{\left( q_{k+1}\delta p_{k+1} + p_{k+1}\delta q_{k+1} \right) } \\&\quad + \sum _{k=0}^{N-1}{\left( D_1H_d^+(q_k,p_{k+1}) \delta q_k + D_2 H_d^+(q_k,p_{k+1}) \delta p_{k+1} \right) } \\&\quad + \sum _{k=0}^{N-1}{\left( \langle \lambda _k ,\nabla \mathcal {C}(q_k) \delta q_k \rangle + \langle \delta \lambda _k , \mathcal {C}(q_k) \rangle \right) } \\&= -\sum _{k=1}^{N-1}{\left( q_{k}\delta p_{k} + p_{k}\delta q_{k} \right) }\\&\quad + \sum _{k=1}^{N-1}{ D_1H_d^+(q_k,p_{k+1}) \delta q_k } + \sum _{k=0}^{N-2}{D_2 H_d^+(q_k,p_{k+1}) \delta p_{k+1}} \\&\quad + \sum _{k=0}^{N-1}{\left( \langle \lambda _k ,\nabla \mathcal {C}(q_k) \delta q_k \rangle + \langle \delta \lambda _k , \mathcal {C}(q_k) \rangle \right) } \\&= -\sum _{k=1}^{N-1}{\left( q_{k}\delta p_{k} + p_{k}\delta q_{k} \right) } + \sum _{k=1}^{N-1}{ D_1H_d^+(q_k,p_{k+1}) \delta q_k } \\&\quad + \sum _{k=1}^{N-1}{D_2 H_d^+(q_{k-1},p_{k}) \delta p_{k}} \\&\quad + \sum _{k=0}^{N-1}{\left( \langle \lambda _k ,\nabla \mathcal {C}(q_k) \delta q_k \rangle + \langle \delta \lambda _k , \mathcal {C}(q_k) \rangle \right) } \\&= \sum _{k=1}^{N-1}{\left[ -q_k +D_2 H_d^+(q_{k-1},p_{k}) \right] \delta p_k } + \sum _{k=0}^{N-1}{ \langle \delta \lambda _k , \mathcal {C}(q_k) \rangle } \\&\quad + \sum _{k=1}^{N-1}{ \left[ -p_k + D_1H_d^+(q_k,p_{k+1}) + \langle \lambda _k, \nabla \mathcal {C}(q_k) \rangle \right] \delta q_k }. \end{aligned}$$

If the discrete constrained right Hamilton’s Eq. (A.14) are satisfied, then each term vanishes and $\delta \mathfrak {S}_d^{+}= 0$. Conversely, if $\delta \mathfrak {S}_d^{+} = 0$, then a discrete fundamental theorem of the calculus of variations yields the discrete constrained right Hamilton’s Eq. (A.14). $\square$

### A.9. Proof of Theorem 3.6

#### Theorem A.9

The Type III discrete Hamilton’s phase space variational principle

A.15 $$\begin{aligned} \delta \mathfrak {S}_d^{-} \left( \{ (q_k,p_k, \lambda _k) \}_{k=0}^{N} \right) = 0 \end{aligned}$$

is equivalent to the discrete constrained left Hamilton’s equations

A.16 $$\begin{aligned}&q_{k} = -D_2 H_d^-(q_{k+1},p_{k}), \qquad p_{k+1} = -D_1H_d^-(q_{k+1},p_{k}) - \langle \lambda _{k+1}, \nabla \mathcal {C}(q_{k+1}) \rangle ,\nonumber \\&\qquad \mathcal {C}(q_k) =0, \end{aligned}$$

where $$H_d^- (q_{k+1},p_{k}) $$ is defined via Eq. (3.17).

#### Proof

Using the fact that $\delta q_N =0$ and $\delta p_0 =0$ since $(q_N,p_0)$ is fixed, we obtain the following expression for the variations of $\mathfrak {S}_d^{-}$:

$$\begin{aligned} \delta \mathfrak {S}_d^-&= \delta \left( - p_0 q_0 - \sum _{k=0}^{N-1}{\left[ -p_{k} q_{k} - H_d^-(q_{k+1},p_{k}) - \langle \lambda _{k+1}, \mathcal {C}(q_{k+1}) \rangle \right] } \right) \\&= \delta \left( \sum _{k=1}^{N-1}{p_{k}q_{k}} + \sum _{k=0}^{N-1}{\left[ H_d^-(q_{k+1},p_{k}) + \langle \lambda _{k+1}, \mathcal {C}(q_{k+1}) \rangle \right] } \right) \\&= \sum _{k=1}^{N-1}{\left( q_{k}\delta p_{k} + p_{k}\delta q_{k} \right) } + \sum _{k=0}^{N-1}{\left( D_1H_d^-(q_{k+1},p_{k}) \delta q_{k+1} + D_2 H_d^-(q_{k+1},p_{k}) \delta p_{k} \right) } \\&\quad + \sum _{k=0}^{N-1}{\left( \langle \lambda _{k+1} ,\nabla \mathcal {C}(q_{k+1}) \delta q_{k+1} \rangle + \langle \delta \lambda _{k+1} , \mathcal {C}(q_{k+1}) \rangle \right) } \\&= \sum _{k=0}^{N}{\left( q_{k}\delta p_{k} + p_{k}\delta q_{k} \right) } + \sum _{k=0}^{N-2}{ D_1H_d^-(q_{k+1},p_{k}) \delta q_{k+1} } + \sum _{k=1}^{N-1}{D_2 H_d^-(q_{k+1},p_{k}) \delta p_{k}} \\&\quad + \sum _{k=0}^{N-1}{\left( \langle \lambda _{k+1} ,\nabla \mathcal {C}(q_{k+1}) \delta q_{k+1} \rangle + \langle \delta \lambda _{k+1} , \mathcal {C}(q_{k+1}) \rangle \right) } \\&= \sum _{k=1}^{N-1}{\left( q_{k}\delta p_{k} + p_{k}\delta q_{k} \right) } + \sum _{k=1}^{N-1}{ D_1H_d^-(q_k,p_{k-1}) \delta q_k } + \sum _{k=1}^{N-1}{D_2 H_d^-(q_{k+1},p_{k}) \delta p_{k}} \\&\quad + \sum _{k=1}^{N}{\langle \lambda _{k} ,\nabla \mathcal {C}(q_{k}) \delta q_{k} \rangle } + \sum _{k=0}^{N-1}{ \langle \delta \lambda _{k+1} , \mathcal {C}(q_{k+1}) \rangle }\\&= \sum _{k=1}^{N-1}{ \left[ q_k + D_2 H_d^-(q_{k+1},p_{k}) \right] \delta p_k } + \sum _{k=0}^{N-1}{ \langle \delta \lambda _{k+1} , \mathcal {C}(q_{k+1}) \rangle } \\&\quad + \sum _{k=1}^{N-1}{ \left[ p_k + D_1H_d^-(q_k,p_{k-1}) + \langle \lambda _k ,\nabla \mathcal {C}(q_k) \rangle \right] \delta q_k }. \end{aligned}$$

If the discrete constrained left Hamilton’s Eq. (A.16) are satisfied, then each term vanishes and $\delta \mathfrak {S}_d^{-} = 0$. Conversely, if $\delta \mathfrak {S}_d^{-} = 0$, then a discrete fundamental theorem of the calculus of variations yields the discrete constrained left Hamilton’s Eq. (A.16). $\square$
